# Correlations of Fecal Metabonomic and Microbiomic Changes Induced by High-fat Diet in the Pre-Obesity State

**DOI:** 10.1038/srep21618

**Published:** 2016-02-26

**Authors:** Hong Lin, Yanpeng An, Fuhua Hao, Yulan Wang, Huiru Tang

**Affiliations:** 1 CAS Key Laboratory of Magnetic Resonance in Biological Systems, State Key Laboratory of Magnetic Resonance and Atomic and Molecular Physics, National Centre for Magnetic Resonance in Wuhan, Wuhan Institute of Physics and Mathematics, University of Chinese Academy of Sciences, Wuhan, 430071, China; 2 State Key Laboratory of Genetic Engineering, Collaborative Innovation Center for Genetics and Development, Ministry of Education Key Laboratory of Contemporary Anthropology, Metabonomics and Systems Biology Laboratory, School of Life Sciences, Fudan University, Shanghai, 200438, China; 3 Collaborative Innovation Center for Diagnosis and Treatment of Infectious Diseases, Zhejiang University, Hangzhou, 310058, China

## Abstract

Obesity resulting from interactions of genetic and environmental factors becomes a serious public health problem worldwide with alterations of the metabolic phenotypes in multiple biological matrices involving multiple metabolic pathways. To understand the contributions of gut microbiota to obesity development, we analyzed dynamic alterations in fecal metabonomic phenotype using NMR and fecal microorganism composition in rats using pyrosequencing technology during the high-fat diet (HFD) feeding for 81 days (pre-obesity state). Integrated analysis of these two phenotypic datasets was further conducted to establish correlations between the altered rat fecal metabonome and gut microbiome. We found that one-week HFD feeding already caused significant changes in rat fecal metabonome and such changes sustained throughout 81-days feeding with the host and gut microbiota co-metabolites clearly featured. We also found that HFD caused outstanding decreases in most fecal metabolites implying enhancement of gut absorptions. We further established comprehensive correlations between the HFD-induced changes in fecal metabonome and fecal microbial composition indicating contributions of gut microbiota in pathogenesis and progression of the HFD-induced obesity. These findings provided essential information about the functions of gut microbiota in pathogenesis of metabolic disorders which could be potentially important for developing obesity prevention and treatment therapies.

Obesity has now become a serious health problem for billions of people in both developed and developing countries across all age and gender groups. The latest data showed that over 30% adults and 16.9% children in US were suffering from obese with the estimated healthcare costs at staggering 100–200 billion dollars every year^1^,^2^. The situation is broadly similar in developing countries. For instance, in China alone, about 200 million people and 12.8% children are obese or overweight with the associated medical costs reaching about 3 billion dollars every year^3^,^4^. Obesity is also known as an important risk factor for dysfunctions of multiple organs leading to metabolic syndrome, nonalcoholic fatty liver disease (NAFLD), cardio-cerebrovascular diseases and even cancers^5^,^6^.

Obesity results from interactions of both genetic and environmental factors causing metabolic alterations in multiple mammalian organs^7^,^8^. It is now well known that *Ob* gene regulates energy balance and its mutation causes regulation dysfunctions of metabolic activities, fat accumulation, body temperature and weight, leading to metabolic diseases such as obesity and diabetes^9^. High-fat diet (HFD) is one of the most studied environmental factors for pathogenesis and progression of obesity, which causes not only fat dispositions but also metabolic alterations in multiple matrices of mammals^7^,^8^. Nevertheless, gut microbiota is now also considered as a vital “environmental” factor for pathogenesis of both genetic and acquired obesity and obesity-related metabolic disorders with the symbiotic interactions with their mammalian hosts as the major attributes^10^. Gut microbiota interactions with HFD are particularly important for obesity and associated metabolic diseases. HFD-induced obesity and Type 2 Diabetes Mellitus (T2DM) are closely associated with alterations in gut microbiota which modulates host metabolism and enhances energy harvest through secreting intestinal peptide hormones, glucagon-like peptide-1 and peptide YY^11^,^12^,^13^. Gut microbiota also modulates the endocannabinoid (eCB) system to regulate intestinal permeability and circulation level of bacterial lipopolysaccharide (LPS)^14^ causing low-grade chronic inflammation in multiple organs and triggering metabolic disorders such as obesity and T2DM^15^.

HFD feeding causes profound changes in gut microbiota contributing to various metabolic disorders through alteration of gut microbiota metabolism and host-microbiota co-metabolisms. The levels of Firmicutes and Bacteroidetes divisions were found to be vastly different for obese and lean subjects in both animal models and human cohort^16^,^17^. HFD also lowered the bacterial abundance in *Lachnospiraceae* and *Ruminococcaceae* families who showed positive correlation with the production of short-chain fatty acids (SCFAs)^18^. In fact, intestinal SCFAs (including formate, acetate, propionate, butyrate and valerate) are microbial metabolites through fermentation of dietary fibers and/or other resistant carbohydrates have multiple functions such as supplying energy to enterocytes, reducing intestinal pH, regulating glycometabolism and inhibiting intestinal inflammation. The HFD-induced level decreases of SCFAs appear to be related to the pathogenesis of metabolic syndrome^19^. Nevertheless, dynamic analysis of microbiome, fecal SCFAs and fecal calories for *ob/ob* and wild-type mice (fed with low-fat diet and HFD) has revealed that relationships between microbiome, energy harvest and obesity are age-dependent and much more complex than anticipated^19^. Furthermore, HFD intervention promotes the generation of bacterial deoxycholate which enhances the gut permeability and increases the risk of liver cancer^20^. Moreover, HFD intake causes elevation of some microbiota metabolites such as trimethylamine oxide (TMAO), indole, benzoate and phenylacetate, which have adverse effects and pathogenic potentials for metabolic and cardiovascular diseases^10^,^21^. However, it remains unknown whether other fecal metabolic activities (other than these) are related to or contributing to the pathogenesis and progression of HFD-induced obesity.

Integrated analysis^12^,^22^ of the fecal metabonomic and microbiomic phenotypes is a potentially feasible way to define the dynamic features of fecal metabolic profiles and their correlations with gut microbiota during obesity development. This is because metabonomics enables systemic detection and quantification of metabolite composition in integrated biological systems and dynamic responses to both endogenous and exogenous perturbation^23^,^24^. In fact, metabonomic analysis has already been successfully applied to multiple biological matrices of animal models for HFD-induced obesity^7^,^8^, T2DM^25^, intestinal bowel diseases^26^ and atherosclerosis^27^. Fecal metabonomic analysis has also been applied in defining the age-dependence of metabolic phenotypes for gastrointestinal contents of rats^28^, IBD (inflammatory bowel disease) related fecal metabolism and diagnosis^29^,^30^, chemical and nutritional effects^22^,^31^,^32^. To the best of our knowledge, however, there is no published report focusing on the dynamic fecal metabolic alteration and the of host-microbiome co-metabolism during the development of HFD-induced obesity. It is also conceivable that HFD-induced changes in gut microbiome will be accompanied with variations fecal metabolic profiles since these fecal metabolites are mostly derived from gut microbiota.

Here, we analyzed the HFD-induced dynamic changes of rat fecal metabonomic phenotypes during 81-days feeding using the NMR-based metabonomics approach and the changes of fecal fatty acid composition using GC-FID/MS techniques. We also analyzed the HFD-induced changes in rat fecal microbiome during such feeding using the pyrosequencing technology and the correlations of the fecal metabonome and microbiome. This study is aimed to understand the HFD-induced dynamic changes in fecal metabonomic phenotypes, gut microbiota and their correlations in the pre-obesity state.

## Results

### Metabolites Detected in Fecal Samples

The average ^1^H-NMR spectra of fecal samples (see Supplementary Fig. S1) for both control and HFD fed rats contained rich metabolite information. The spectral signals were unambiguously assigned based on the publically accessible and in-house developed databases^22^,^28^. Both the ^1^H and ^13^C NMR signals of 59 fecal metabolites were further confirmed (see Supplementary Table S1) with a series of 2D NMR spectra including amino acids, carbohydrates, nucleotides, organic bases, organic acids, gut microbiota-related metabolites such as SCFAs. Visual inspection of these spectra (see Supplementary Fig. S1) revealed that the levels of fecal phenylalanine and tyrosine were higher but these of niacin, hypoxanthine and uridine diphosphate glucose (UDPG) were lower for HFD-fed rats than controls. Amongst fecal fatty acids (see Supplementary Table S2), palmitic acid (C16:0), stearic acid (C18:0) and their unsaturated forms (C16:1, C18:1 and C18:2) dominated the long-chain fatty acids (LCFAs) whilst C22:1 and C24:0 were two major very long-chain fatty acids (VLCFAs).

### HFD-induced Fecal Metabonomic Changes

PCA (Principal Components Analysis) revealed some outliers at various time points which were removed during the subsequent analyses; the results indicated that obvious fecal metabonomic differences were probably present between the HFD and control groups. The corresponding OPLS-DA (Orthogonal Partial Least Squares Discriminant Analysis) models all showed good qualities judged from the Q^2^ values (0.86–0.95) (see Supplementary Fig. S2) and the results of permutation tests and CV-ANOVA (see Supplementary Table S3). This further confirmed the presence of significant fecal metabonomic differences between the HFD and control groups at all these sampling time points. The metabolic trajectories derived from PCA results showed two distinct diet-induced dynamic features for the HFD and control groups (see Supplementary Fig. S3).

**Figure 1:**
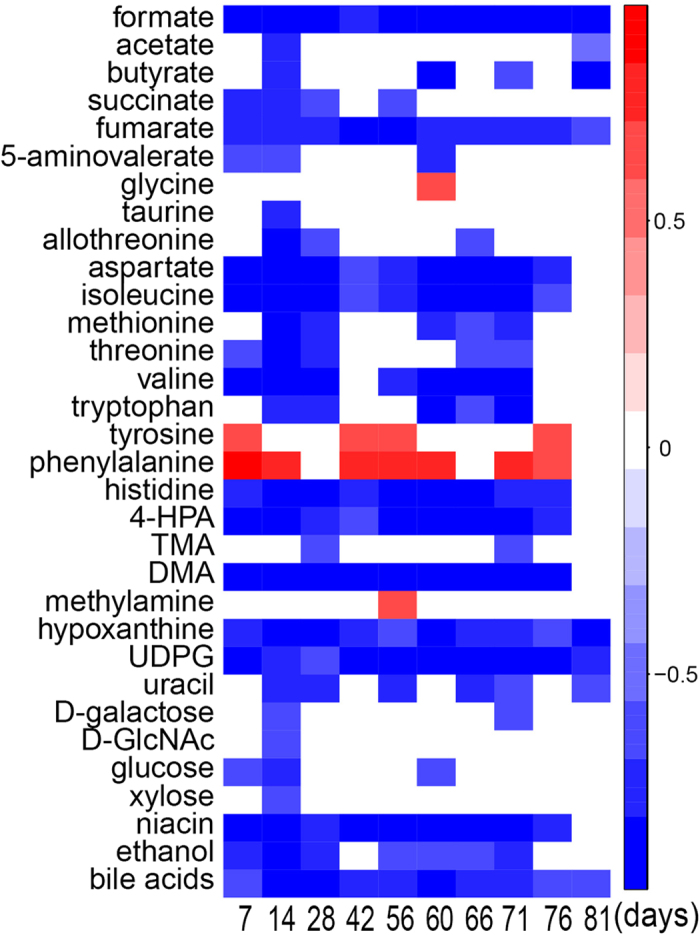
HFD-induced dynamic changes in the fecal metabonome against controls with the Pearson correlation coefficients from OPLS-DA color-coded. The red cells indicated the HFD-induced significant metabolite level increases whereas blue ones indicated decreases. UDPG: uridine diphosphate glucose; TMA: trimethylamine; DMA: dimethylamine; D-GlcNAc: N-Acetyl-D-glucosamine; 4-HPA: 4-hydroxyphenylacetate.

OPLS-DA results further showed that about 33 fecal metabolites had significant level differences between the HFD and control groups throughout 81-days treatment (Fig. 1, Table 1). Fecal tyrosine and phenylalanine levels were significantly higher in HFD group compared with the controls whereas HFD feeding caused significantly decreases for many other fecal amino acids (taurine, allothreonine, isoleucine, threonine, valine, 5-aminovalerate, tryptophan, histidine), short chain fatty acids (formate, acetate, butyrate), purines and pyrimidines (hypoxanthine, uridine diphosphate glucose, uracil), niacin, hexoses (galactose, glucose, xylose), N-acetyl-D-glucosamine (D-GlcNAc), TCA cycle intermediates (fumurate, succinate), bile acids and ethanol (Fig. 1, Table 1). HFD also induced significant level decreases for some gut microbiota related metabolites including 4-hydroxyphenylacetate (4-HPA), trimethylamine (TMA) and dimethylamine (DMA) (Fig. 1, Table 1). Furthermore, the ratios of HFD-induced metabolite changes were plotted as a function of feeding durations to indicate the dynamic scales of such changes (see Supplementary Fig. S4). Compared with control diet, HFD feeding caused more than 40% level reduction for His, uridine diphosphate glucose (UDPG), 4-HPA, formate and hypoxanthine. HFD also induced about 10–40% level declines for most fecal amino acids but about 20–40% level increases for two aromatic amino acids, phenylalanine and tyrosine (see Supplementary Fig. S4). After HFD feeding for 28 and 56 days, levels of most LCFAs were significantly elevated whereas VLCFAs and C18:2 significantly decreased. It is also interesting to note that levels of most of the detectable saturated fatty acids have shown significant increases from 4- to 8-weeks control-diet feeding but no changes for those fatty acids primarily from microbes such as C15:0 and C19:0 (see Supplementary Table S2).

### High-fat diet Caused Changes in Gut Microbiota Structure

To reveal the HFD effects on gut microbiota structure, we analyzed the microbial composition of fecal samples at day 7, 28, 56 and 81 after high-fat diet treatment. The richness and diversity^33^ of fecal microbial composition in both the HFD and control groups fluctuated over the feeding period (see Supplementary Fig. S5). The richness of gut microbiota reflected as total OTU numbers was higher in HFD group than in controls though with statistical significance only at day-7 (*p* = 0.005) and day-56 (*p* = 1.5E-06) (see Supplementary Fig. S5a). Fecal microbial diversity, indicated as Shannon index, had some tendency of increases in the HFD group with significant inter-group differences only at day-56 (*p* = 0.001) (see Supplementary Fig. S5b). Firmicutes, Bacteroidetes, Tenericutes and Proteobacteria were dominant microbial divisions. The relative abundance of Firmicutes was increased but that of Bacteroidetes decreased in both the HFD-fed rats and controls at all time points. HFD-fed rats had significant higher abundance of Firmicutes but lower abundance of Tenericutes than controls after 4-weeks treatment (see Supplementary Fig. S6) whereas abundance of Bacteroidetes for the HFD-fed rats was lower than controls after 8-weeks treatment (see Supplementary Fig. S6c). For both groups, the Firmicutes-to-Bacteroidetes ratio in terms of their relative abundance increased steadily with prolonged feeding although such ratios were significant higher in the HFD group than in controls after 8-weeks feeding (see Supplementary Fig. S6d).

**Figure 2:**
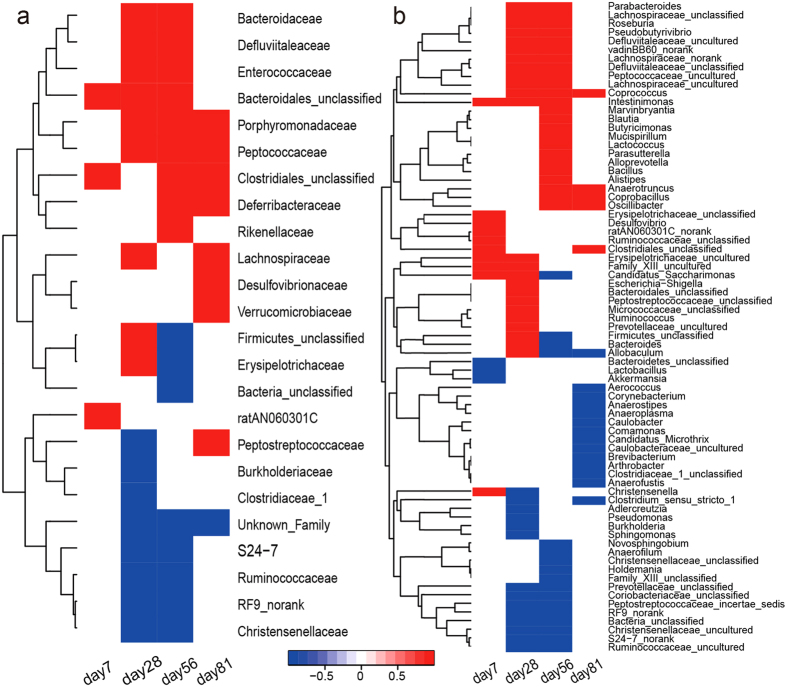
HFD-induced significant gut microbial changes against controls in the (a) family and (b) genus levels, respectively, at day 7, 28, 56 and 81 post treatments. The changes at each time-point were color-coded with values of 1-p where p-values were from the Student’s t-test or Kruskal-Wallis test. The red cells indicated the HFD-induced significant elevations of microbes whereas the blue ones indicated decreases. The white cells indicate the microbes with no significant inter-group differences.

In the family levels, HFD feeding induced comprehensive and significant alterations of gut microbiota structure (Fig. 2). After only one-week feeding, HFD already caused significant abundance elevation for specific families of Clostridiales and Bacteroidales whereas the abundance of many more families were increased such as Bacteroidaceae, Enterococcaceae and Peptococcaceae from 4-weeks onwards accompanied with abundance decreases for Clostridiaceae, Ruminococcaceae and Christensenellaceae (Fig. 2a). HFD-caused significant abundance changes are also clearly highlighted in the genus level (Fig. 2b). One-week HFD feeding led to elevation of *Intestinimonas, Christensenella, Desulfovibrio, Candidatus_Saccharimonas* and some unclassified genera but abundance declines for *Lactobacillus, Akkermansia* and some unclassified genus from Bacteroidetes (Fig. 2b). After HFD feeding for 4-weeks onwards, HFD-induced abundance microbial changes were observed for many more genera (Fig. 2b). In the species levels, HFD feeding for 7 days caused significant abundance changes for dozens microbial species and such changes became more significant by prolonged HFD treatment with about 72 species showing significant changes during 81-days HFD treatment (see Supplementary Fig. S7, Table S4).

### Correlation between the HFD-induced Changes in Fecal Metabolites and Gut Microbiota

**Figure 3:**
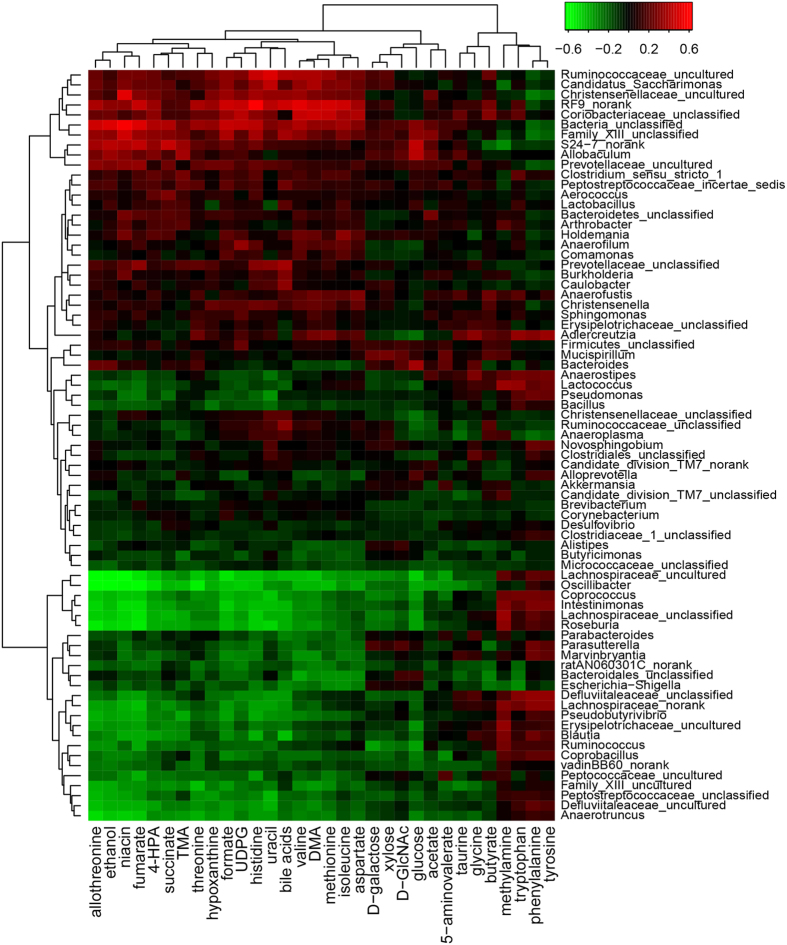
Correlations for the significantly changed fecal metabolites and microbial genera with the respective hierarchical clustering. The correlation coefficients between specific fecal metabolites and certain bacterial genera were color-coded with hot color (e.g., red) denoting positive correlations whereas the cool one (e.g., green) indicating negative ones. 4-HPA: 4-Hydroxyphenylacetate; TMA: Trimethylamine; DMA: Dimethylamine; UDPG: Uridine diphosphate glucose; D-GlcNAc: N-Acetyl-D-glucosamine.

To investigate the interactive features between fecal metabolites and gut microbiota during the HFD-induced obesity development, the clustering correlations were analyzed between the HFD-induced changes in fecal metabolites and gut microbial abundance in family, genus and species levels (Fig. 3, Table 2, 3, 4, 5, see Supplementary Fig. S8,S9, Table S5). The HFD-induced changes of fecal metabolites were clustered together broadly according to their involvement in metabolic pathways. Such changes also showed comprehensive correlations with the HFD-induced changes in gut microbes in all three levels with red color indicating positive correlations whereas green denoting negative correlations. In the family level (see Supplementary Fig. S8), fecal monosaccharides showed positive correlation with *Deferribacteraceae* but negative ones with *Peptococcaceae, Defluviitaleaceae* and *Lachnospiraceae* (Table 2). Three SCFAs (formate, acetate and butyrate) showed negative correlation with *Bacteroidales*; formate and acetate also showed negative correlations with unclassified family in *Clostridiales*, *Defluviitaleaceae* and *Porphyromonadaceae* whereas butyrate further showed positive correlation with *Clostridiaceae* and *Peptostreptococcaceae* but negatively with *Rikenellaceae* (Table 3). Three aromatic amino acids (tyrosine, phenylalanine and tryptophan) and three methylamines (TMA, DMA and MA) in feces had negative correlations with specific bacteria in *Bacteroidales* although DMA and TMA also showed negative correlations with *Defluviitaleaceae, Peptococcaceae* and *Porphyromonadaceae* (Tables 4 and 5). Bile acids were positively correlated with *Ruminococcaceae* but negatively with *Deferribacteraceae, Defluviitaleaceae, Porphyromonadaceae* and *Bacteroidales_unclassified* (see Supplementary Table S5).

Fecal metabolites further showed correlations with microbes in genus level (Fig. 3). Fecal monosaccharides showed negative correlations with *Coprococcus, Ruminococcus, Roseburia* and some specific bacteria in *Lachnospiraceae* (Table 2). Three fecal aromatic amino acids had positive correlations with *Adlercreutzia, Anaerostipes, Coprococcus* and some unidentified genera in *Defluviitaleaceae* and *Lachnospiraceae* (Table 4); Phe and Tyr had positive correlations with *Intestinimonas*. TMA and DMA showed negative correlations with *Coprococcus, Intestinimonas, Pseudobutyrivibrio, Roseburia* and a unclassified genus in *Lachnospiraceae* family whereas MA had positive correlations with them (Table 5). TMA and DMA also showed negative correlations with *Anaerotruncus* and some uncultured genera from *Defluviitaleaceae, Family_XIII,* and *Lachnospiraceae* families (Table 5). Bile acids were positively correlated with *Burkholderia, Caulobacter, RF9* and some unidentified or uncultured genera in *Bacteria, Christensenellaceae, Family_XIII, Peptostreptococcaceae, Prevotellaceae,* and *Ruminococcaceae* but negatively with *Anaerotruncus, Blautia, Coprobacillus, Coprococcus, Escherichia-Shigella, Intestinimonas, Oscillibacter, Parabacteroides, Pseudobutyrivibrio, Roseburia,* and some uncultured or unclassified genera in *Defluviitaleaceae, Erysipelotrichaceae, Lachnospiraceae* families (see Supplementary Table S5).

In the species level, moreover, fecal metabonome showed outstandingly complex correlations with gut microbial species detected (see Supplementary Fig. S9) and those with statistical significance were tabulated. Fecal monosaccharides (glactose, xylose and glucose) correlated negatively with *Anaerotruncus sp. G3, Clostridium papyrosolvens, Lachnospiraceaebacterium_3_1_57FAA_CT1, Lactococcus plantarum,* and 12 uncultured or unclassified species (Table 2) whereas SCFAs showed negative correlations with *Allobaculum stercoricanis* and a unclassified *Parvibacter* species (Table 3). Three fecal aromatic amino acids (Phe, Tyr and Trp) had positive correlations with *Blautia glucerasea, Lactococcus plantarum, Lachnospiraceae_bacterium_3_1_57FAA_CT1,* and 6 uncultured or unclassified species (Table 4). TMA and DMA were correlated negatively with *Acidovorax wohlfahrtii, Butyricimonas virosa* and 5 unclassified species which were inversely positively correlated with MA (Table 5). Bile acids were positively correlated with *Clostridium_sp._Culture-27, Parasutterella_secunda, Turicibacter_sp._LA61* and 11 unclassified species but negatively with *Acidovorax wohlfahrtii, Allobaculum stercoricanis, Anaerotruncus_sp._G3, Bacteroides uniformis, Blautia glucerasea, Butyricimonas virosa, Clostridium papyrosolvens, Enterococcus gallinarum, Microbacterium maritypicum, Lachnospiraceae_bacterium_3_1_57FAA_CT1, Lactococcus plantarum,* and 26 unclassified species (see Supplementary Table S5).

## Discussion

Previous study reported that 81-day HFD feeding led to development of rat obesity accompanied with changes in metabolic phenotypes of animals especially some mammal and gut microbiota co-metabolisms^7^. Such HFD feeding also caused increases in intestinal permeability by down-regulating the genes encoding tight junction proteins on the mucosal epithelial cells^34^. In this work, we discovered that HFD feeding induced significant changes in fecal metabolic profiles and gut microbiota structure for rat obesity development, both of which correlated comprehensively. Such fecal metabonomic changes were detectable as early as a week after high-fat diet treatment and sustained throughout 81-day HFD intake. These changes involved with amino acids and metabolites, TCA cycle intermediates, carbohydrates, short-chain fatty acids (SCFAs), LCFAs, VLCFAs, metabolites of gut microbiota from choline (TMA, DMA, MA) and tyrosine (4-HPA). This indicates that gut microbiota profiles and their metabolic profiles (including fatty acids) are all associated with the pathogenesis of the HFD-induced rat obesity.

First, our results showed that during 81-day HFD feeding, rat fecal metabonomic profiles had consistent and sustained changes suggesting that HFD-induced profound changes in metabolic activities of gut microbiota during obesity development. Fecal metabolites are originated from intestinal epithelial cells, gut microbes, microbial metabolism of indigestible dietary substances, and host-microbial co-metabolisms though the microbe-related ones are dominant since metabolites from dietary sources are absorbed completely in small intestine. For example, amino acids, fatty acids (LCFAs, VLCFAs), TCA cycle intermediates, nucleotides are mostly endogenous metabolites of gut microbes because most of such metabolites from diets are absorbed in small intestine^28^. 2-Ketoisovalerate and 4-HPA are well-known bacterial metabolites of valine and tyrosine, respectively, whilst 5-aminovalerate is a microbial metabolite of lysine or D-proline^12^. TMA, DMA and MA are microbial metabolites of choline^35^ whereas most fecal bile acids are the bacterial-derived secondary metabolites^36^. D-GlcNAc is probably from bacterial cell walls whereas fecal xylose and galactose are normally degradation products of dietary hemicellulose (e.g., xylan, galactan, arabinan and glycan) catalyzed by the gut bacterial hydrolytic enzymes since these dietary fibers are indigestible for mammals due to lack appropriate degradation enzymes^37^,^38^. These monosaccharides further undergo colonic fermentation producing SCFAs such as acetate, propionate and butyrate^39^,^40^. Such monosaccharides are important source of energy for certain intestinal bacteria and SCFAs can be absorbed by intestinal epithelium cells as well.

HFD feeding also induced significant changes in gut microbiota structure especially reflected in the microbial abundance. Differing from previous results for well established obesity^13^,^41^, our results showed that the species-level richness and diversity of fecal microbe composition in both the HFD and control groups fluctuated over the feeding period (see Supplementary Fig. S5) with higher richness of gut microbiota in HFD group than in controls only at day-7 and day-56 and significant inter-group diversity differences only at day-56. This is probably because animals in this study were in the pre-obese state and only reached obesity level after HFD feeding for 12 weeks. In fact, bacterial diversity in species level was also fairly constant for obesity human cohort during a weight losing process with two calorie restricted diets^17^. Nevertheless, HFD-induced abundance changes for two dominant bacterial divisions, Firmicutes and Bacteroidetes (see Supplementary Fig. S6), were broadly agreeable with the trends in previous reports for obese mice and human^11^,^16^,^17^. We also observed significant lower abundance of Tenericutes in HFD-fed rats than controls after 4-weeks HFD treatment which was not reported previously. The Firmicutes-to-Bacteroidetes ratio in terms of their abundance were significant higher in the HFD group than in controls after 8-weeks feeding (see Supplementary Fig. S6) although such ratios in both groups showed a trend of increase over the feeding period. These changes are probably associated with the HFD-induced pathogenesis of obesity.

HFD-induced comprehensive and significant alterations of gut microbiota structure are also observable in bacterial family levels (Fig. 2). Following only one-week feeding, HFD has already caused significant abundance elevation for Clostridiales and Bacteroidales (Fig. 2a). After HFD feeding for 4-weeks, the abundance of more families such as Bacteroidaceae, Enterococcaceae and Peptococcaceae were elevated with concurrent abundance decreases for Clostridiaceae, Ruminococcaceae and Christensenellaceae (Fig. 2a). In genus level, such HFD-induced changes were also comprehensive (Fig. 2b). HFD has caused significant abundance elevation for *Candidatus, Saccharimonas, Christensenella, Desulfovibrio, Intestinimonas*, and some unidentified genera in Erysipelotrichaceae, Erysipelotrichaceae, Family_XIII, Ruminococcaceae, ratAN060301C and Clostridiales after only one-week feeding (Fig. 2b). The abundance of more genera microbes were elevated after HFD feeding 4-weeks including *Coprococcus, Intestinimonas, Parabacteroides, Pseudobutyrivibrio, Roseburia* and some unidentified genera in Defluviitaleaceae, Defluviitaleaceae, Lachnospiraceae, Peptococcaceae and vadinBB60 with concurrent abundance decreases for unidentified genera in *Christensenellaceae, Coriobacteriaceae, Peptostreptococcaceae, Prevotellaceae, RF9, Ruminococcaceae*, and *S24-7* (Fig. 2b). In the species level, over 70 microbial species showed significant abundance changes during 81-days HFD treatment, many of them were not yet identifiable so far (see Supplementary Fig. S7, Table S4). Nevertheless, HFD feeding for as short as 7 days already caused significant abundance changes for dozens microbial species and such changes had an obvious dependence on the feeding durations; the HFD-induced alterations were more marked with prolonged HFD treatment (see Supplementary Fig. S7, Table S4).

Moreover, correlations were observable between the HFD-induced changes in fecal metabolites and gut microbial (Fig. 3) providing interactive functional information associated with HFD-cause obesity. Such microbiome-metabonome correlations were present between human urinary metabonome and gut microbiome^12^, antibiotics treated animal models^22^,^42^, and genetic obese^10^. In fact, previous metagenomic analysis of human distal samples revealed that intestinal microbiome involved in a number of their host metabolic processes including production, transportation and metabolism of polysaccharides, lipids, amino acids, peptides, nucleotides, vitamins, bile acids and xenobiotics^43^. HFD-induced changes for the levels of D-galactose (Gal), D-glucose (Glc), D-xylose (Xyl), and D-GlcNAc were clustered together and had similar variation patterns during 81-days feeding (Fig. 3, Table 2). Their changes showed inverse correlations with the abundance changes of microbes in family Lachnospiraceae and genera *Ruminococcus* whereas Gal, Glc and Xyl also had inverse correlations with genera *Coprococcus* and *Roseburia* (Fig. 3, Table 2). This is understandable since all microbes in family Lachnospiraceae, genera *Ruminococcus, Coprococcus* and *Roseburia* are important promoters for production of SCFAs^44^,^45^. Although these monosaccharide-consuming bacteria were expected to have positive correlations with the levels of intestinal SCFAs, no obvious relationship were observed in the correlation analysis between dynamically changed fecal metabolites and altered bacteria (Fig. 3, Table 3). This is because SCFAs have multiple functions as an energy source for the colonic epithelium cells, inter- and intra-cellular pH regulators and the modulators of host cellular signal transduction by interacting with relevant receptors^46^. Recent studies showed that SCFAs activated orphan G protein-coupled receptor 43 leading to suppression of the insulin signaling in adipocytes and fat accumulation in adipose tissue^47^. Lower levels of monosaccharides and SCFAs in the HFD group than in controls in this study were probably due to much reduced contents of carbohydrates in high-fat diet employed in our study^7^.

The elevation of fecal phenylalanine and tyrosine caused by HFD feeding together with decline of tryptophan (Fig. 1) observed here were possibly related to HFD-induced changes of these gut bacteria having metabolic functions towards these aromatic amino acids. Three aromatic acids (Phe, Tyr and Trp) were closely clustered together and their changes showed strong positive correlations with the changes of gut bacteria in *Adlercreutzia*, *Anaerostipes*, *Coprococcus* genera from Lachnospiraceae family (Fig. 3, Table 4). In fact, such correlations were much complex with phenylalanine having positive correlations with five genera bacteria but inverse correlations with three. Tyr correlated positively with two genera whereas tryptophan showed positive correlations with one genera but inverse correlations with two (Fig. 3, Table 4).

It is well known that tyrosine, phenylalanine and tryptophan are essential amino acids for mammals and can be degraded in intestine by gut microbiota. Phenylalanine is converted to phenyllactate and then phenylpropionate by some species of *Clostridium*^48^. Phenylalanine can also be converted into tyrosine which is further metabolized anaerobically into 4-HPA and then *p*-cresol^48^. Tryptophan is converted into indolepropionate by tryptophanase produced by *Clostridia* bacteria^48^.

Fecal TMA, DMA and methylamine (MA) were mostly choline metabolites from gut microbiota. TMA is often re-absorbed into host liver and converted into trimetlylamine-oxide by flavine monooxygenase as a detoxification pathway^39^. HFD-induced level reduction of fecal TMA (Fig. 1) probably resulted from its enhanced transportation to liver supported by observed TMA elevation in this group of the HFD fed animals^7^. In this work, fecal TMA level showed positive correlations with the bacteria in genera *Allobaculum* and *Clostridium* whilst DMA had positive correlations with bacteria in *Adlercreutzia*, *Anaerofustis*, *Candidatus Saccharimonas*, and some uncultured genera in Christensenellaceae, Coriobacteriaceae, Ruminococcaceae, and RF9 families; MA had positive correlations with bacteria in *Blautia*, *Coprococcus*, *Lactococcus*, *Pseudobutyrivibrio*, *Roseburia*, *Ruminococcus, Intestinimonas* and some unconfirmed genera in Erysipelotrichaceae, Lachnospiraceae, Lachnospiraceae (Fig. 3, Table 5). This is agreeable with the reported findings that *Clostridium perfringens* is important producer for these methylamines in mammalian intestine^40^.

Fecal bile acids showed comprehensive correlations with many microbial genera by showing positive correlation with *Burkholderia* and *Caulobacter* together with some genera from Christensenellaceae, Peptostreptococcaceae and Prevotellaceae but negative ones with genera *Anaerotruncus, Blautia, Coprobacillus, Coprococcus, Parabacteroides, Pseudobutyrivibrio, Roseburia* and *Intestinimonas* (Fig. 3, see Supplementary Table S5). It is well known that *Bacteroides* are major contributors to deconjugation of bile acids in intestine whereas *Clostridium* are important contributors for producing secondary bile acids^22^. Fecal taurine and glycine were clustered together in the correlation analysis and positively correlated with genera *Adlercreutzia* while negatively correlated with *Family_XIII* and *Oscillibacter* (Fig. 3). However, it remains unknown whether such correlations are related to the metabolic functions of these microbes towards bile acids especially for these microbes yet to be identified unambiguously. Since metabolism of bile acids and contributions of gut microbiota towards such metabolic processes are remarkably complex, quantitative correlations between all bile acid species and microbial classes clearly warrant further detailed investigation especially in the pathogenesis of obesity. This work is currently ongoing.

To sum up, our metabonomic and microbiomic analyses revealed that HFD feeding induced dynamic changes in the fecal metabonomic phenotypes and gut microbiota composition in the pre-obesity state. Such changes were detectable well before reaching the obesity state (i.e., one and four weeks after HFD intakes) and comprehensive correlations were observable between rat fecal metabonome and microbiome. HFD-induced fecal metabonomic changes were highlighted by level changes in fecal aromatic amino acids and their microbial metabolites, monosaccharides from microbial hydrolysis of dietary fibers, SCFAs derived from microbial fermentation of these carbohydrates, organic amines derived from microbial metabolism of choline, TCA intermediates, bile acids and nucleotides. HFD-induced abundance changes were detectable for many microbes which were well known for their functions in biotransformations of the above metabolites. Further analysis found comprehensive correlations between the HFD-induced changes in fecal metabolites and bacterial abundances showing comprehensive microbe-metabolite relationships. These findings not only offered important information on the microbial functions in the development of obesity even in the pre-obesity state which might inspire potential development of obesity prevention methods, but also suggested necessity for further exploration of the detailed intestinal bacterial metabolisms related to obesity development and prevention. This work also showed the necessity for developing novel strategies to unambiguously identify these microbial species which had corrections with fecal metabolites.

## Materials and Methods

### Chemicals

Chemicals were purchased from commercial sources as detailed in Supplementary Information. Phosphate buffer (0.1 M, pH = 7.40) containing 30% D_2_O (v/v), NaN_3_ 0.03% (w/v) and TSP 0.002% (w/v) was prepared with K_2_HPO_4_ and NaH_2_PO_4_ and employed as extracting solvent for fecal metabolites with its low-temperature stability^49^.

### Sample Collection

Animal samples were from the same experiment described previously^7^. Fecal samples for every animal were collected into individual 5 mL eppendorf tubes from all rats (n = 12) the day before HFD intervention and every week during HFD feeding so that samples were traceable to each animal and each time point. The samples were snap-frozen with liquid nitrogen and stored at −80 °C until further analysis.

### Sample Preparation for NMR and GC-FID/MS Analysis

A previously optimized method for fecal sample preparation for NMR analysis was adapted^50^. Fecal fatty acids were quantified as their methyl esters using the previously reported methods^27^,^51^ with some minor modifications. These details are deposited in the Supplementary Information. Moisture contents were measured gravimetrically for every fecal sample as described previously^28^.

### NMR Analysis of Fecal Extracts

Details for NMR spectra acquisition are described in Supplementary Information. A set of two-dimensional NMR spectra was acquired for some selected samples with acquisition and processing parameters as reported previously^52^.

### NMR Data Processing and Multivariate Data Analysis

NMR data were processed as described in the Supplementary Information and the integrated areas of all bins were normalized to the dry sample weights so that the resultant data represented the absolute concentration of bins (or metabolites) in the form of peak area per milligram dry sample.

Multivariate data analysis was conducted on the normalized data using the software SIMCA-P+ (V12.0, Umetrics, Sweden). Principal component analysis (PCA) was performed with the mean-centered data (unless stated otherwise) to generate an overview of group clustering and to detect possible outliers. The orthogonal projection to latent structure discriminant analysis (OPLS-DA) was conducted with the unit-variance scaling and 7-fold cross-validation^53^ to obtain metabolites having significant inter-group differences. Qualities of OPLS-DA models were further assessed with CV-ANOVA^54^ with *p* < 0.05 as significant. After back-transformation, loadings were plotted using an in-house developed script with the correlation coefficients of all variables color-coded, where the warm colored variables contributed more to inter-group differences than the cool ones. The significantly differentiated metabolites were extracted based on the Pearson correlation coefficients in the level of *p* < 0.05 to generate a heatmap using MATLAB 7.1 software to visualized the significant changed metabolites as a function of treatment duration^53^,^54^.

The ratios of concentration changes for metabolites were calculated individually against their concentration in control group and expressed as (C_H_−C_C_)/C_C_, where C_H_ and C_C_ stood for the average concentrations of a metabolite in HFD and control groups respectively.

### DNA Extraction, PCR Amplification, Pyrosequencing and Bioinformatics Analysis

Fecal samples from day 7, day 28, day 56 after HFD feeding were employed for microbiome analysis using 454 pyrosequencing technology as described previously^55^. Bacterial DNA was extracted from about 100 mg rat feces using E.Z.N.A.**^®^** Stool DNA Kit (Omega Bio-Teks HiBind technology, Norcross, GA) and amplified with 533R (5^′^-TTACCGCGGCTGCTGGCAC-3^′^) as forward primer and 27F (5^′^-AGAGTTTGATCCTGGCTCAG-3^′^) as the reverse primer specifically for V1-V3 regions of the 16S rRNA. The PCR analysis was conducted on a thermocycler PCR system (ABI GeneAmp PCR system 9700, USA) using 20 μl TransStart Fastpfu DNA Polymerase. The PCR products were then sequenced on a 454 Life Sciences Genome Sequencer FLX system (Roche, Basel, Switzerland). All the operation procedures above were according to the manufacturer’s instructions.

The raw pyrosequencing reads were denoised with Titanium PyroNoise software and filtered using Qiime (version 1.17 http://qiime.org/)^56^. Then removing sequences with average quality score <20 over a 50 bp sliding window and sequences shorter than 200 bp, with homopolymers longer than six nucleotides, and those containing ambiguous base calls or incorrect primer sequences. Operational Taxonomic Units (OUT) were clustered at 97% nucleotide similarity level using Uparse (version 7.1 http://drive5.com/uparse/)^57^ and aligned using RDP classifier (version 2.2 http://sourceforge.net/projects/rdp-classifier/)^58^ at 97% similarity level compared with database of Silva (Release 115 http://www.arb-silva.de)^59^. The alpha-diversity (Shannon and Simpson index) and community richness (Chao and ACE) were calculated with corresponding estimators (http://www.mothur.org)^33^. The correlation heatmaps between fecal metabolites and gut microbiota were generated with gplots package of R software (3.1.2).

## Additional Information

**How to cite this article**: Lin, H. *et al.* Correlations of Fecal Metabonomic and Microbiomic Changes Induced by High-fat Diet in the Pre-Obesity State. *Sci. Rep.* **6**, 21618; doi: 10.1038/srep21618 (2016).

## Supplementary Material

Supplementary Information

## Figures and Tables

**Table 1:** HFD-induced significant changes of fecal metabolites against controls and corresponding correlation coefficients at various days post-treatment (5-AV: 5-aminovalerate; 4-HPA: 4-hydroxyphenylacetate; TMA: trimethylamine; DMA: dimethylamine; UDPG: uridine diphosphate glucose; D-GlcNAc: N-acetyl-D-glucosamine).

metabolites	day7	day14	day28	day42	day56	day60	day66	day71	day76	day81
formate	−0.951	−0.958	−0.951	−0.768	−0.952	−0.973	−0.956	−0.928	−0.874	−0.851
acetate	—	−0.732	—	—	—	—	—	—	—	−0.561
butyrate	—	−0.785	—	—	—	−0.871	—	−0.674	—	−0.911
succinate	−0.743	−0.838	−0.708	—	−0.700	—	—	—	—	—
fumarate	−0.774	−0.763	−0.786	−0.867	−0.915	−0.793	−0.830	−0.758	−0.731	−0.713
5-AV	−0.712	−0.692	—	—	—	−0.802	—	—	—	—
glycine	—	—	—	—	—	0.614	—	—	—	—
taurine	—	−0.789	—	—	—	—	—	—	—	—
allothreonine	—	−0.860	−0.671	—	—	—	−0.594	—	—	—
aspartate	−0.864	−0.935	−0.903	−0.710	−0.837	−0.916	−0.864	−0.918	−0.716	—
isoleucine	−0.858	−0.947	−0.911	−0.585	−0.834	−0.898	−0.846	−0.88	−0.594	—
methionine	—	−0.902	−0.804	—	—	−0.755	−0.622	−0.776	—	—
threonine	−0.650	−0.850	−0.762	—	—	—	−0.648	−0.687	—	—
valine	−0.846	−0.924	−0.854	—	−0.808	−0.889	−0.858	−0.865	—	—
tryptophan	—	−0.717	−0.827	—	—	−0.848	−0.710	−0.886	—	—
tyrosine	0.681	—	—	0.630	0.638	—	—	—	0.586	-
phenylalanine	0.901	0.791	—	0.750	0.752	0.715	—	0.749	0.688	—
histidine	−0.812	−0.919	−0.920	−0.781	−0.864	−0.933	−0.863	−0.767	−0.751	—
4-HPA	−0.920	−0.889	−0.809	−0.707	−0.863	−0.914	−0.925	−0.883	−0.768	—
TMA	—	—	−0.589	—	—	—	—	−0.695	—	—
DMA	−0.885	−0.959	−0.914	−0.856	−0.925	−0.964	−0.944	−0.946	−0.876	—
methylamine	—	—	—	—	0.618	—	—	—	—	—
hypoxanthine	−0.779	−0.887	−0.86	−0.757	−0.665	−0.952	−0.83	−0.736	−0.636	−0.863
UDPG	−0.854	−0.838	−0.691	−0.866	−0.936	−0.958	−0.889	−0.936	−0.968	−0.802
uracil	—	−0.764	−0.804	—	−0.776	—	−0.755	−0.620	—	−0.691
D-galactose	—	−0.678	—	—	—	—	—	−0.685	—	—
D-GlcNAc	—	−0.626	—	—	—	—	—	—	—	—
glucose	−0.624	−0.795	—	—	—	−0.614	—	—	—	—
xylose	—	−0.635	—	—	—	—	—	—	—	—
niacin	−0.932	−0.895	−0.835	−0.863	−0.921	−0.974	−0.849	−0.861	−0.753	—
ethanol	−0.734	−0.905	−0.782	—	−0.649	−0.709	−0.679	−0.768	—	—
bile acids	−0.693	−0.876	−0.870	−0.791	−0.781	−0.855	−0.769	−0.814	−0.657	−0.675

**Table 2:**
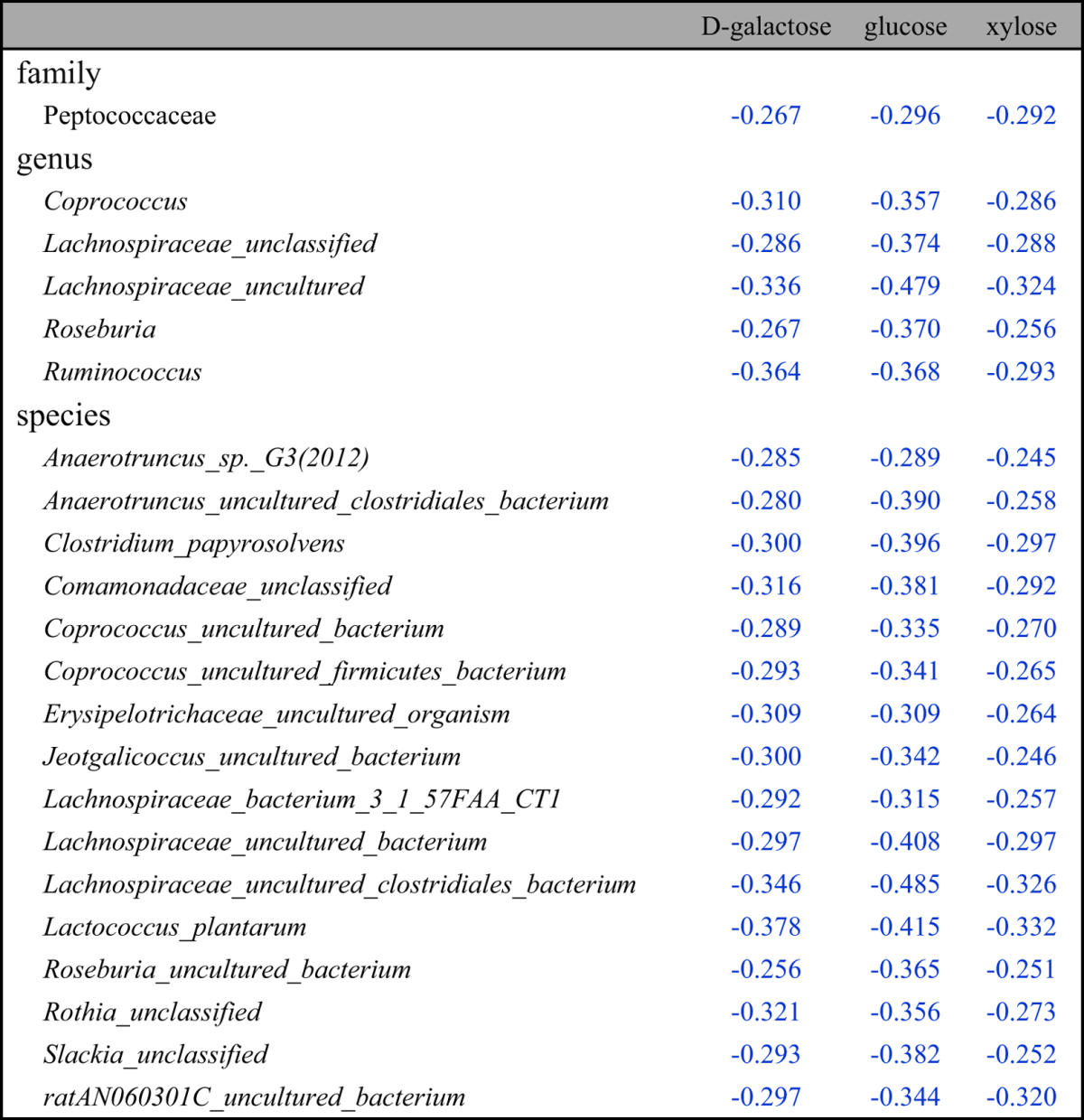
Significant correlations between the HFD-induced changes of fecal monosaccharides and of microbes in the family, genus and species levels.

(Pearson correlation coefficients with red ones indicated positive correlations whereas the blue ones indicated negative correlations; the listed correlation coefficients were these with *p* value < 0.05).

**Table 3:**
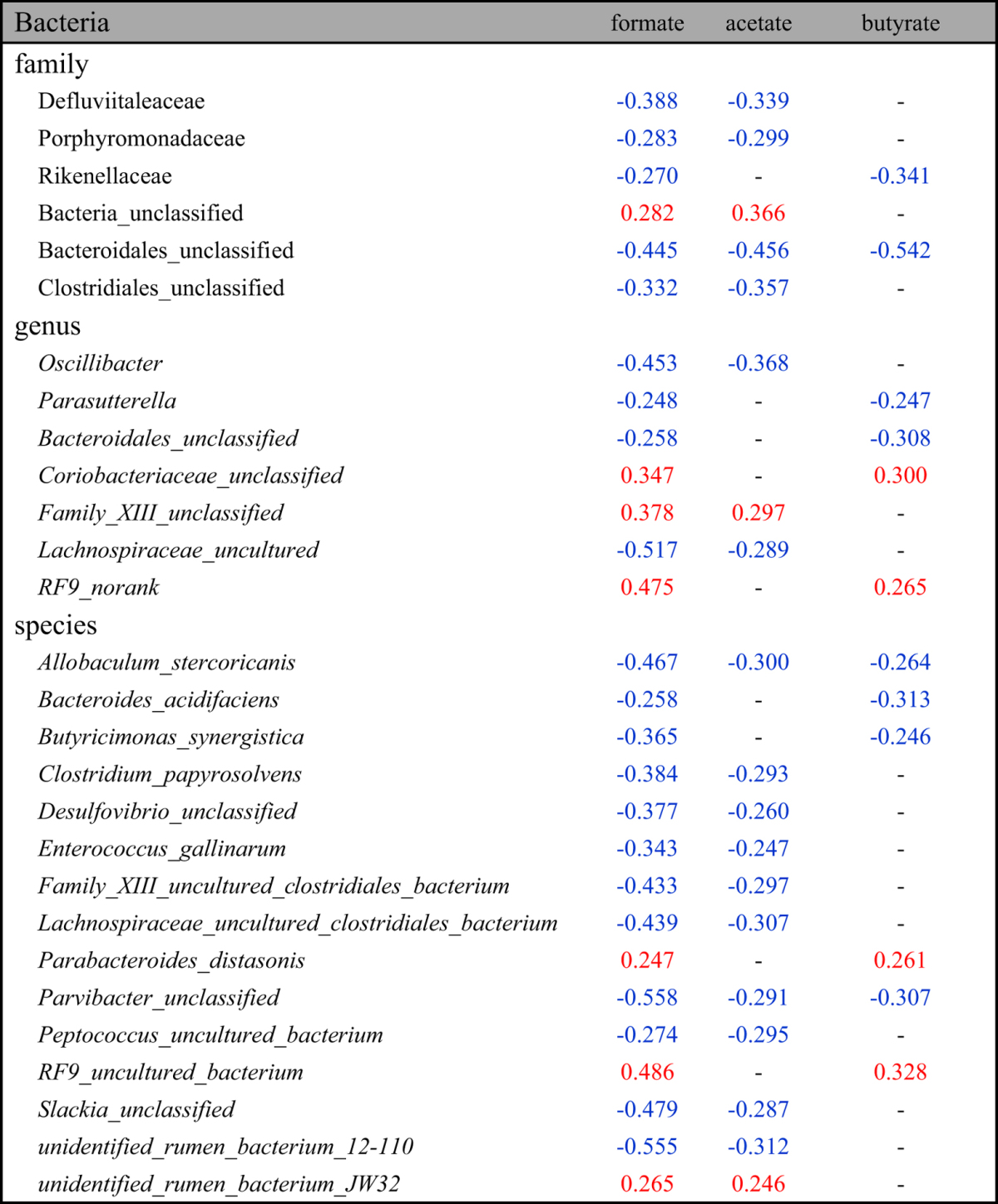
Significant correlations between the HFD-induced changes of fecal SCFAs and of microbes in the family, genus and species levels.

(Pearson correlation coefficients with red color indicated positive correlations whereas these with blue indicated negative correlations; the listed correlation coefficients were these with *p* value < 0.05).

**Table 4:**
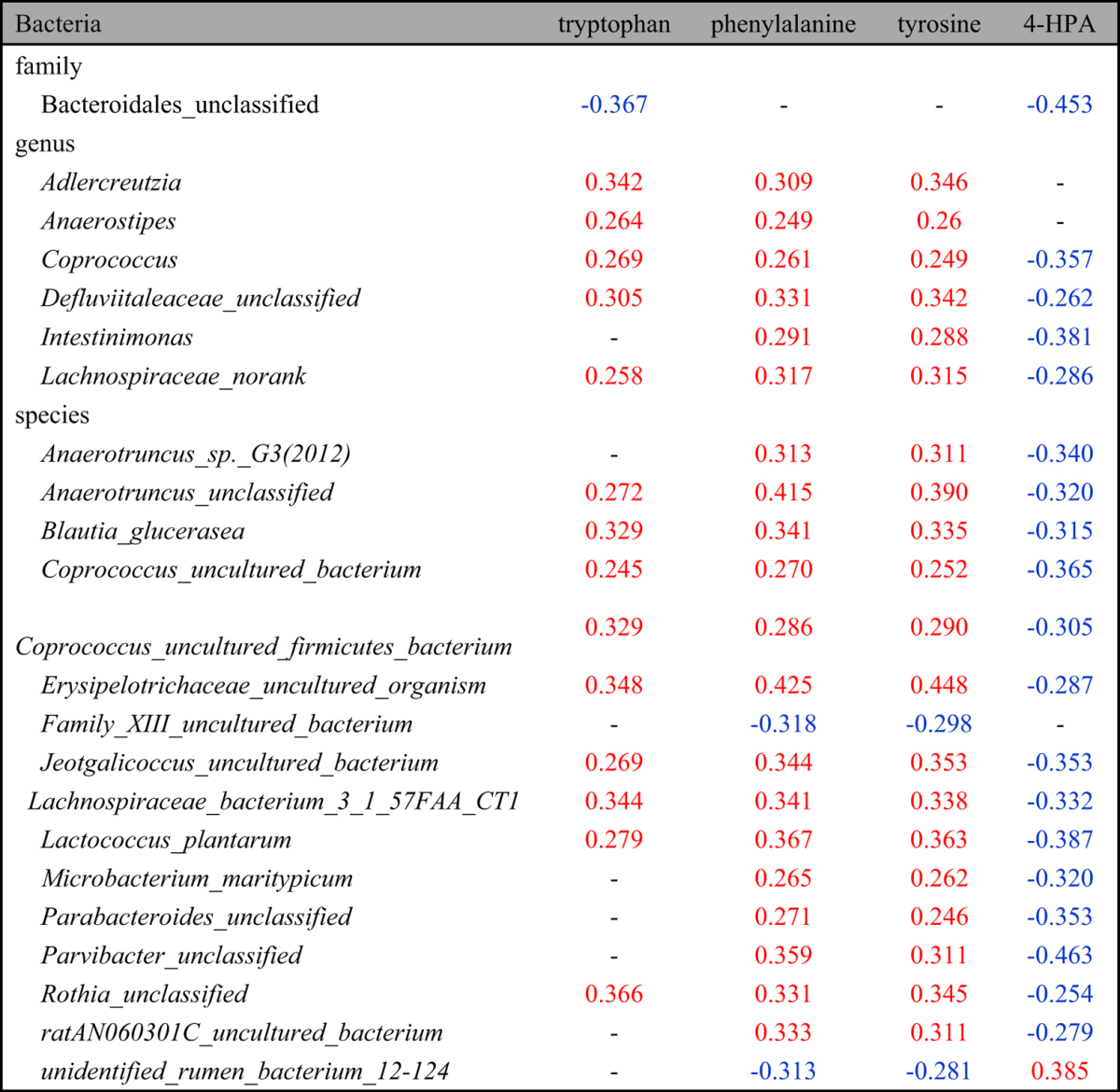
Significant correlations between the HFD-induced changes of fecal metabolites of aromatic amino acids and changes of microbes in the family, genus and species levels.

(Pearson correlation coefficients with red color denoted positive correlations whereas blue ones meant negative correlations; the listed correlation coefficients were these with *p* < 0.05; 4-HPA: 4-Hydroxyphenylacetate).

**Table 5:**
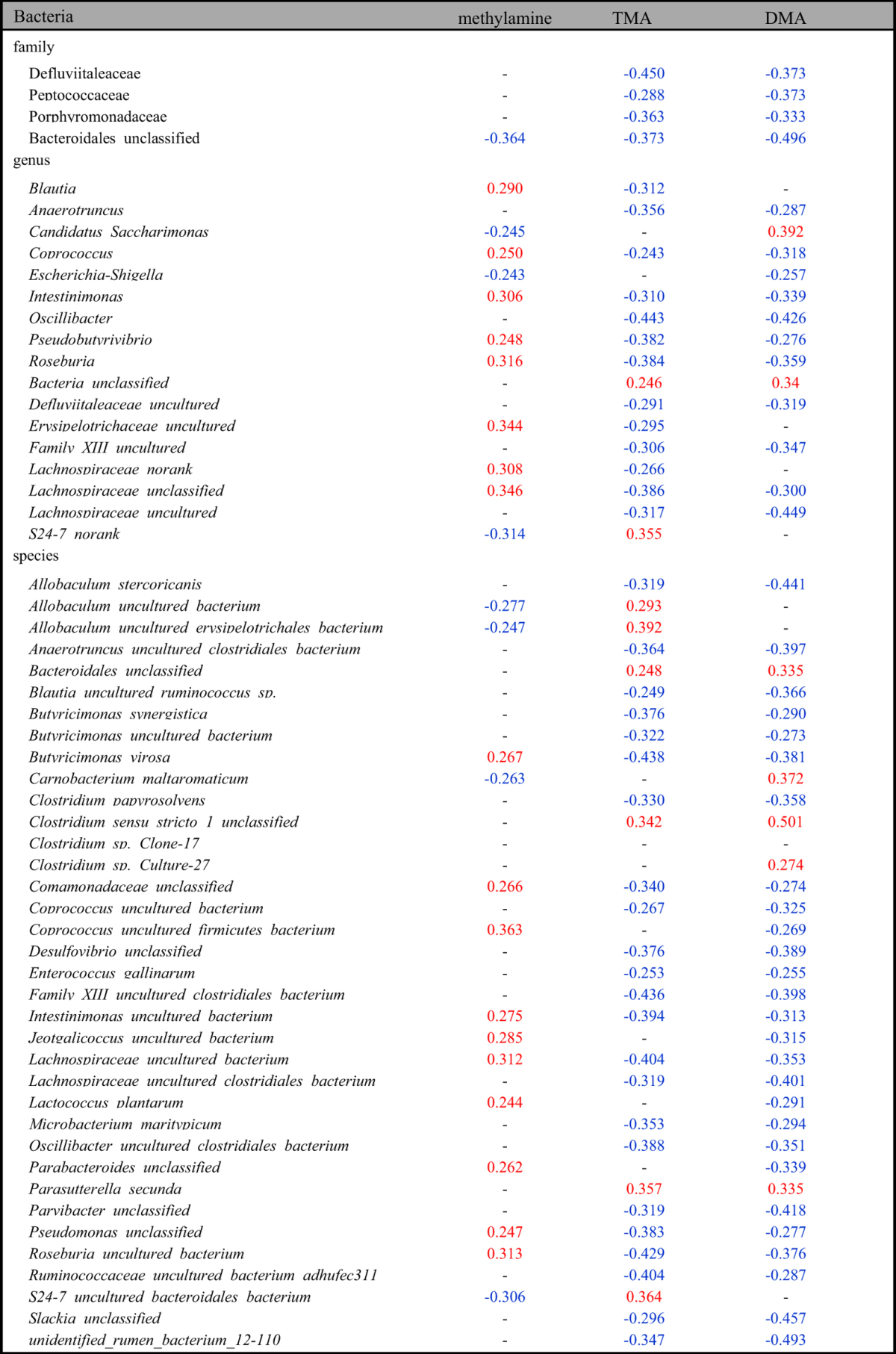
Significant correlations between the HFD-induced changes of fecal methylamines and of microbes in the family, genus and species levels.

(Pearson correlation coefficients in red indicated positive correlations whereas these in blue indicated negative correlations; the listed correlation coefficients were these with *p* < 0.05).
