# Supplementary material for: Correlations of Fecal Metabonomic and Microbiomic Changes Induced by High-fat Diet in the Pre-Obesity State
Source: Sci Rep. 2016 Feb 26;6:21618. doi: 10.1038/srep21618 (PMC4768318; doi:10.1038/srep21618)
Supplement: Supplementary Information [file srep21618-s1.pdf]

Supplementary data

Correlations of Fecal Metabonomic and Microbiomic Changes Induced by High-fat Diet in the Pre-Obesity State

Hong Lin[†#], Yanpeng An[‡#], Fuhua Hao[†], Yulan Wang[†,§], and Huiru Tang[‡*]

[†] *CAS Key Laboratory of Magnetic Resonance in Biological Systems, State Key Laboratory of Magnetic Resonance and Atomic and Molecular Physics, National Centre for Magnetic Resonance in Wuhan, Wuhan Institute of Physics and Mathematics, University of Chinese Academy of Sciences, Wuhan, 430071, China*

[‡] *State Key Laboratory of Genetic Engineering, Collaborative Innovation Center for Genetics and Development, Ministry of Education Key Laboratory of Contemporary Anthropology, Metabonomics and Systems Biology Laboratory, School of Life Sciences, Fudan University, Shanghai, 200438,China*

[§] *Collaborative Innovation Center for Diagnosis and Treatment of Infectious Diseases, Zhejiang University, Hangzhou, 310058, China*

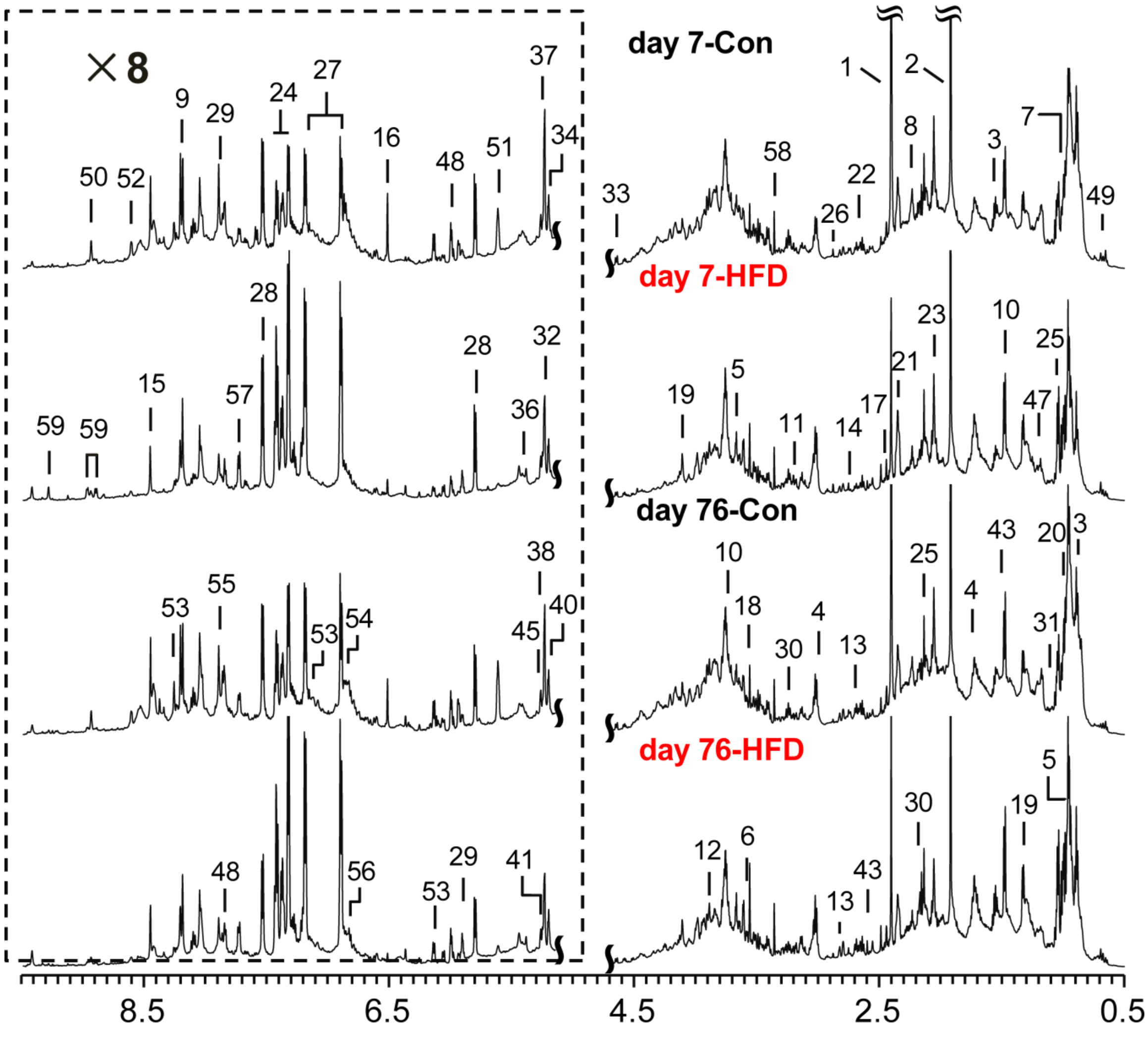

Supplementary Figure S1. Average ^{1}H NMR spectra of fecal samples in control and HFD groups at day 7 and day 76 after treatment. The dotted area were vertically magnified 8 times. The metabolites were numbered and listed in Table S1 (Supporting Information)

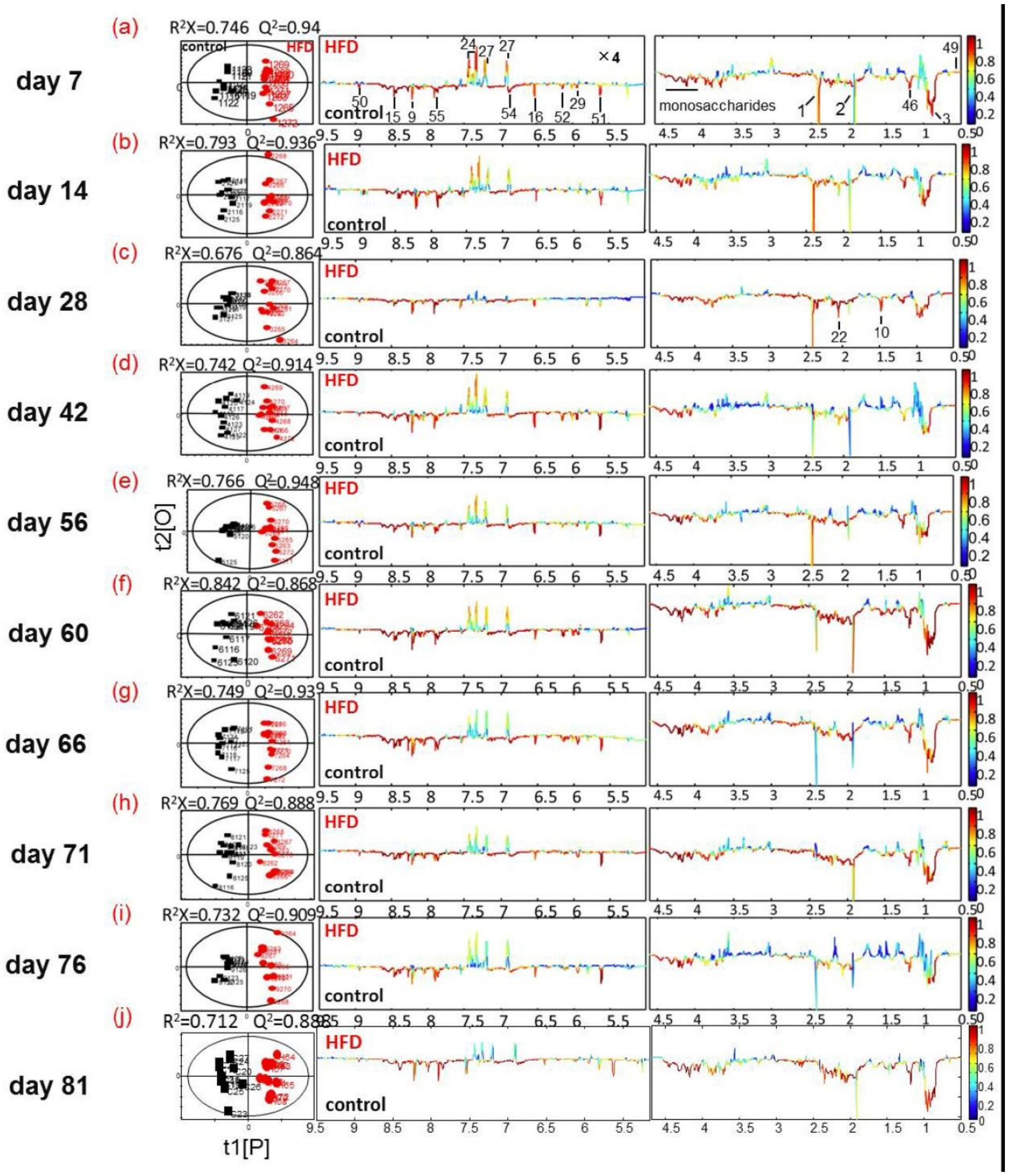

Supplementary Figure S2. OPLS-DA scores plots (left) and corresponding coefficient plots (right) for ^{1}H NMR spectra of fecal water obtained from the HFD (red circle) and control (black squares) groups at several time points: (a) day 7; (b) day 14; (c) day 28; (d) day 42; (e) day 56; (f) day 60; (g) day 66; (h) day 71; (i) day 76; (j) day 81. Metabolite keys are shown in Table S1.

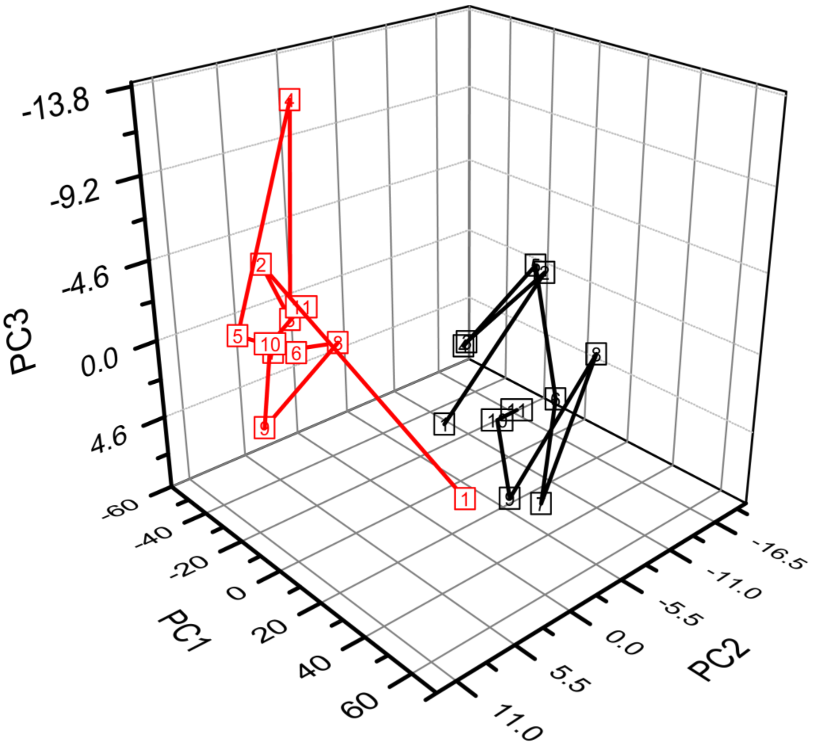

Supplementary Figure S3. 3D fecal metabonomic trajectory during dietary intervention derived from PCA using unit variance scaled data from two groups at (1)day 0, (2)day 7, (3)day 14, (4)day 28, (5)day 42, (6)day 56, (7)day 60, (8)day 66, (9)day 71, (10)day 76, (11)day 81. Each point represents the mean scores of three principal components at a given time point.

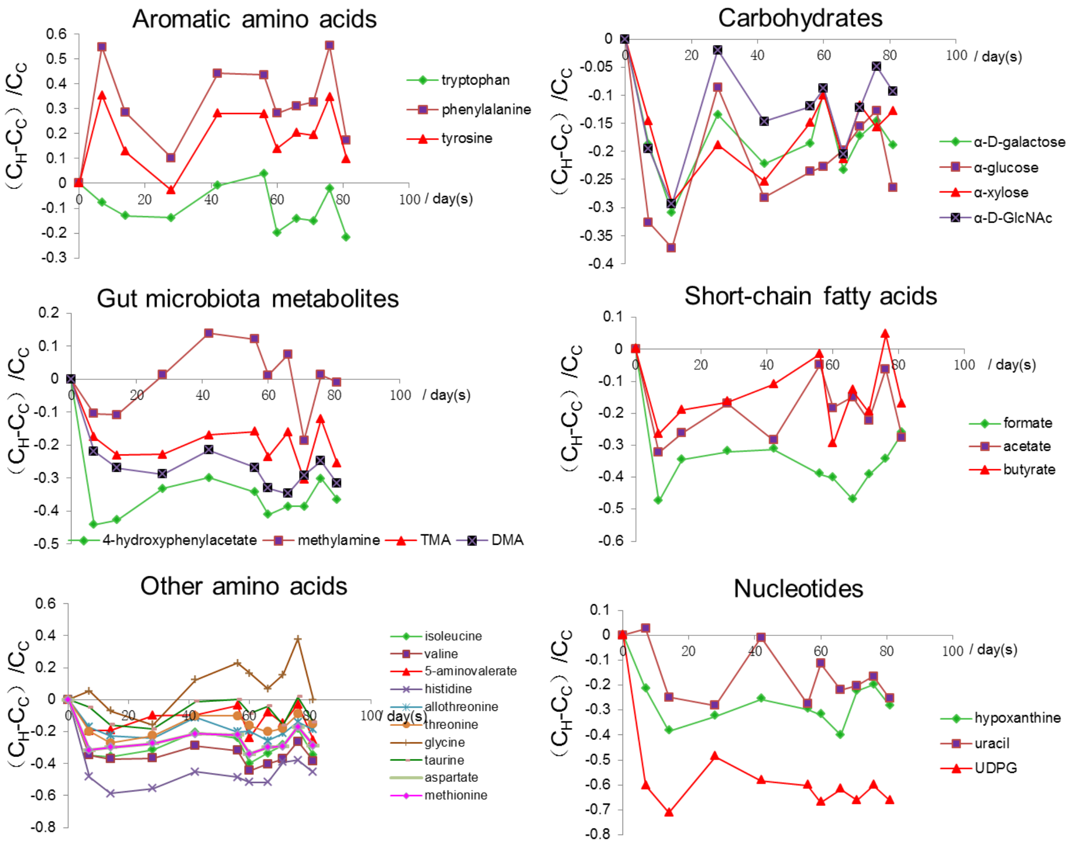

Supplementary Figure S4. The ratios of the HFD-induced concentration changes for fecal metabolites compared with controls. The ratios were expressed as $(C_H-C_C)/C_C$, where C_H and C_C stood for the average concentrations of a metabolite in HFD and control groups respectively.

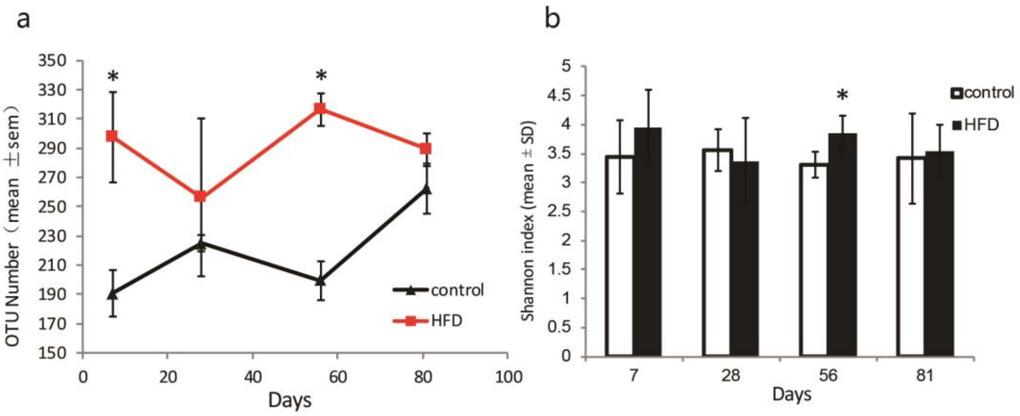

Supplementary Figure S5. The richness (a) and diversity(b) of gut microbiota at four time points after dietary intervention (day 7, day 28, day 56, day 81). *p< 0.05 by the Student t-test or Kruskal-Wallis test.

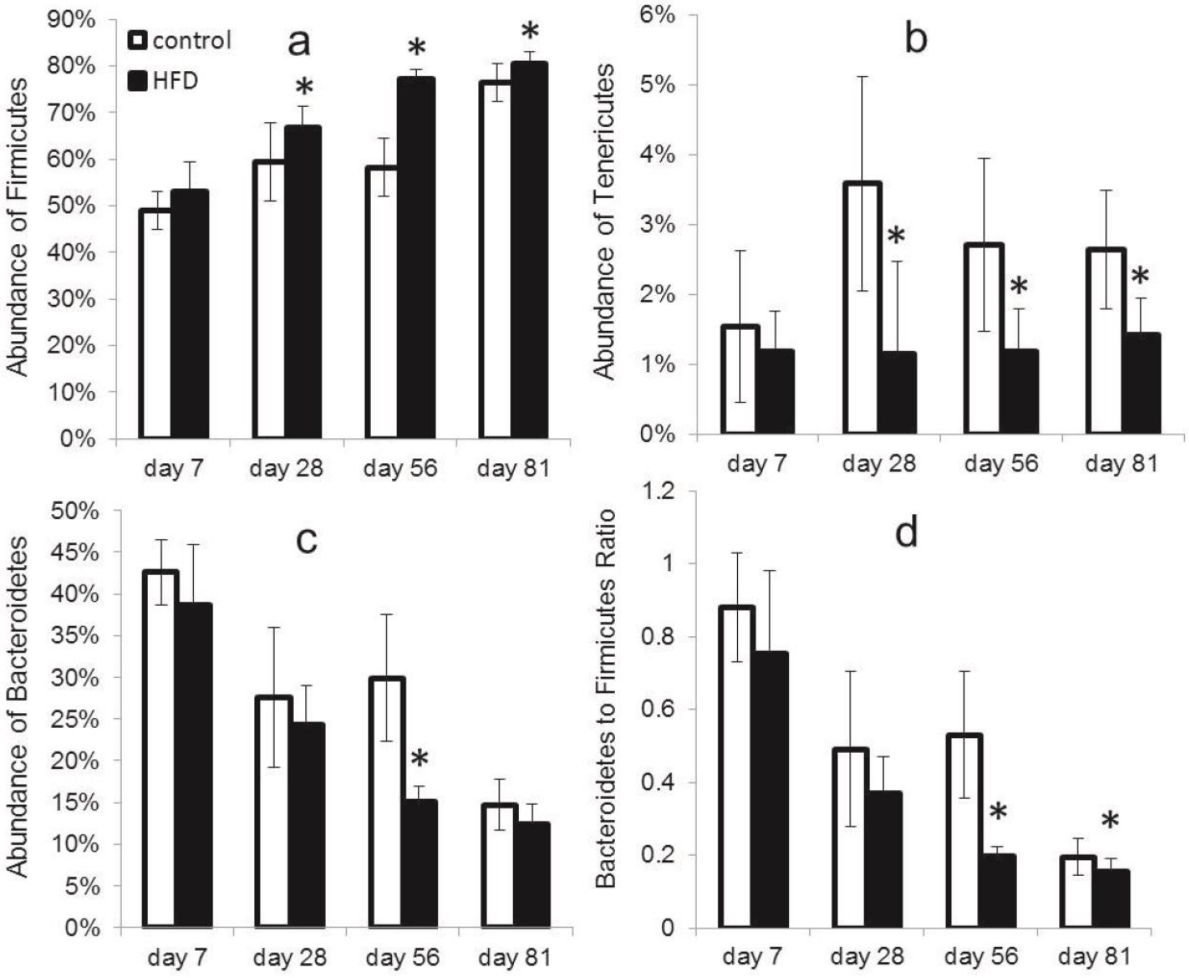

Supplementary Figure S6. The relative abundance of (a) Firmicutes, (b) Tenericutes, (c*)* Bacteroidetes and (d) the Firmicutes-to-Bacteroidetes ratio. *$p < 0.05$, by Student t-test or Kruskal-Wallis test and error bar represents the SD.

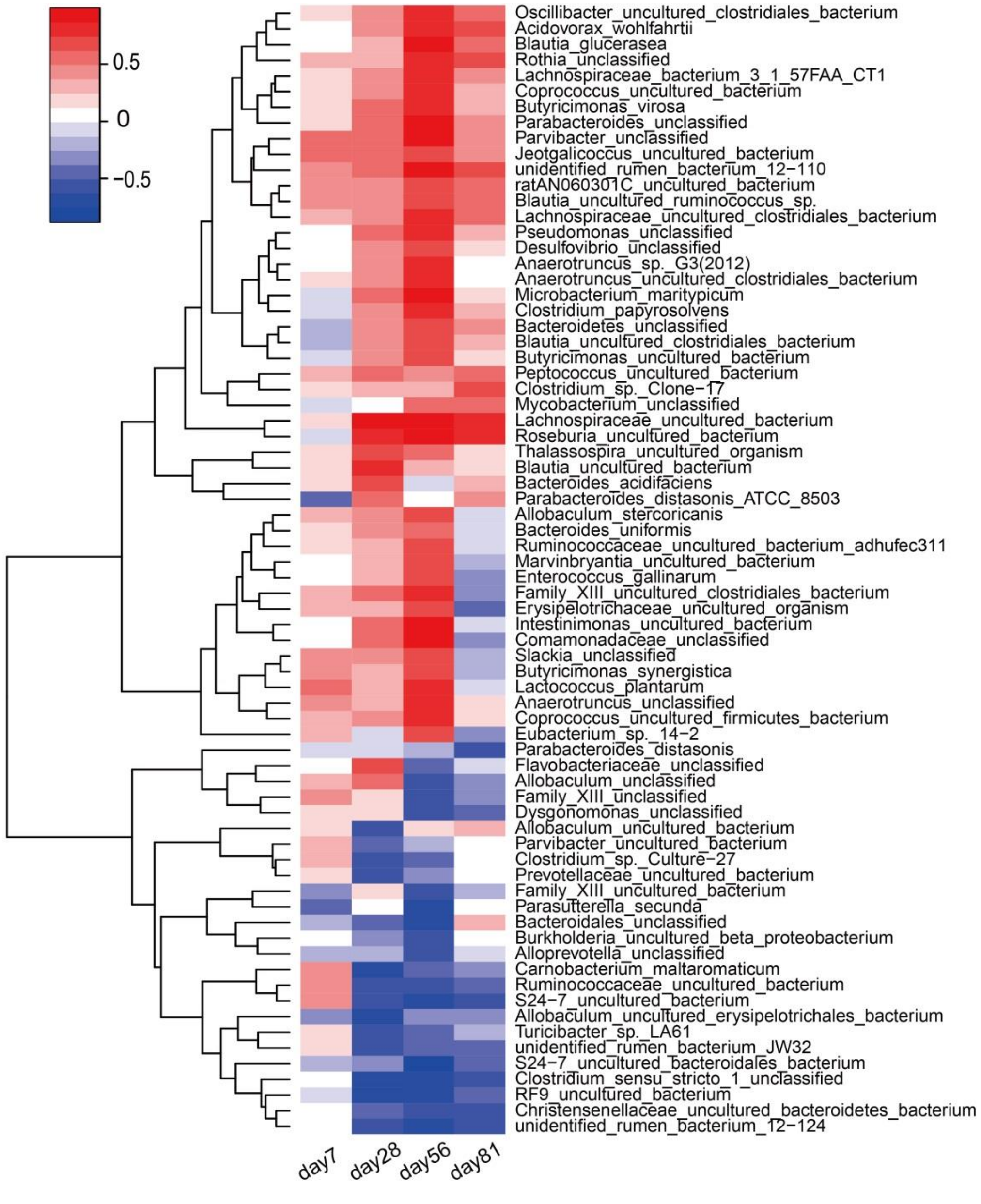

Supplementary Figure S7. The Heat map described the dynamic structural change of species-level community compared HFD group with the control. Red cells indicate the increasing of the bacteria, while blue cells indicate decreasing. The changes of bacteria with more intense colour are more significant.

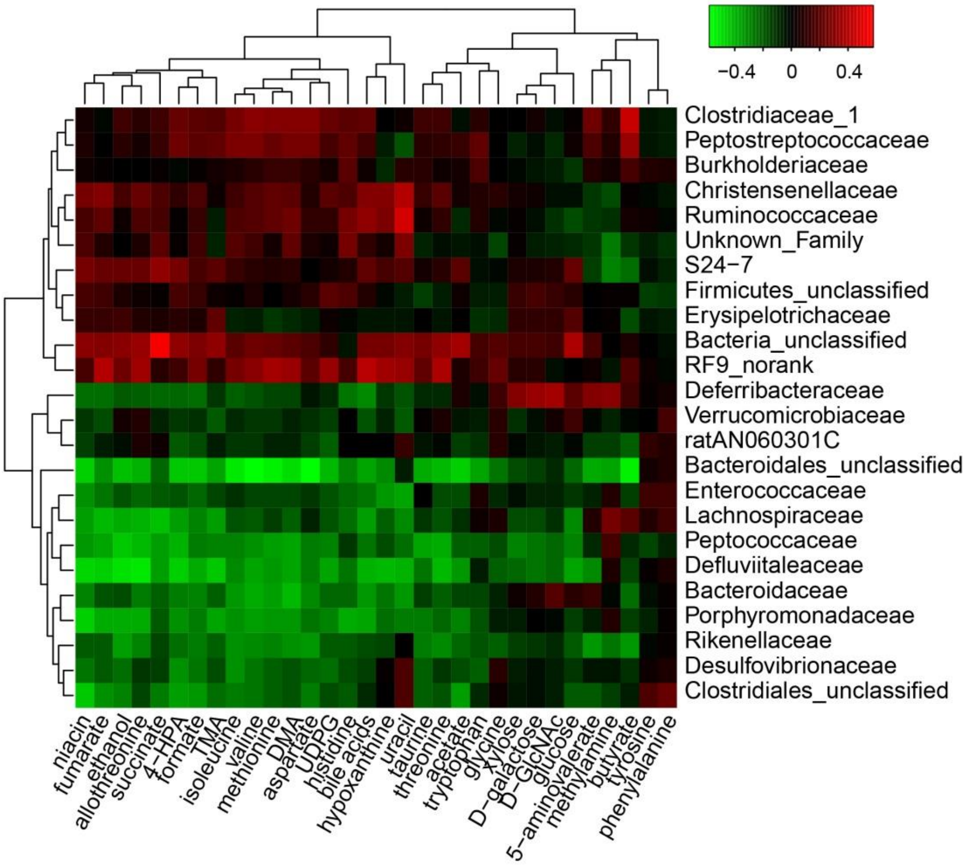

Supplementary Figure S8. Heatmap describing correlations between the HFD-induced significant changes of metabolites and of microbes in the family level with respective hierarchical clustering. The colored blocks represent the correlation coefficients with hot and cold color indicating positive and negative correlations respectively. 4-HPA: 4-Hydroxyphenylacetate; TMA: Trimethylamine; DMA: Dimethylamine; UDPG: Uridine diphosphate glucose; D-GlcNAc: N-Acetyl-D-glucosamine.

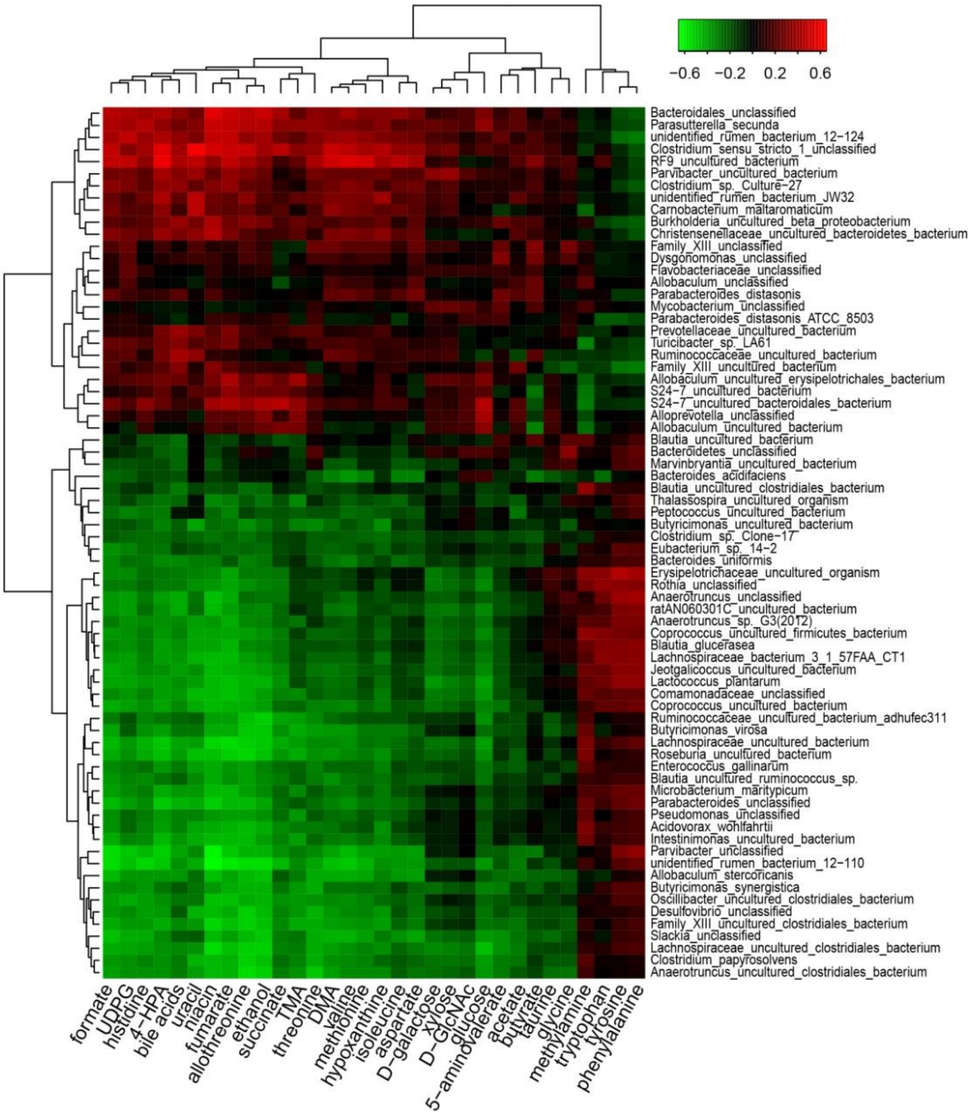

Supplementary Figure S9. Heatmap describing correlations between the HFD-induced significant changes of metabolites and of microbes in the species level with respective hierarchical clustering. The colored blocks represent the correlation coefficients with hot and cold color indicating positive and negative correlations respectively. 4-HPA: 4-Hydroxyphenylacetate; TMA: Trimethylamine; DMA: Dimethylamine; UDPG: Uridine diphosphate glucose; D-GlcNAc: N-Acetyl-D-glucosamine.

Supplementary Table S1. Assignments for NMR signals of fecal metabolites

No.	Metabolites	Group	δ ^{1}H (multiplicity)	δ ^{13}C
1	Succinate	CH_3	2.41(s)[a]	36.88
		COO^-		184.61
2	Acetate	CH_3	1.92(s)	26.01
		COO^-		184.19
3	Butyrate	αCH_2	2.16(t)	42.45
		βCH_2	1.56(sextet)	22.06
		γCH_3	0.90(t)	16.12
		COOH		186.81
4	Lysine	αCH	3.77(t)	57.22
		βCH_2	1.91(m)	32.62
		γCH_2	1.48(m)	24.07
		δCH_2	1.73(m)	29.16
		εCH_2	3.03(t)	41.87
		COO^-		177.23
5	Isoleucine	αCH	3.68(d)	62.37
		βCH	1.98(m)	38.62
		γCH_3	1.01(d)	17.41
		γCH_2	1.27(m)	27.32
		γCH_2	1.47(m)	27.32
		δCH_3	0.94(t)	13.91
		COO^-		176.98
6	Threonine	αCH	3.59(d)	63.22
		βCH	4.26(dd)	68.74
		γCH_3	1.33(d)	22.23
		COO^-		177.05
7	Valine	αCH	3.62(d)	63.25
		βCH	2.28(m)	31.86
		γCH_3	1.04(d)	20.72
		γCH_3	0.99(d)	19.41
		COO^-		177.04
8	5-Aminovalerate	αCH_2	2.24(t)	39.58
		βCH_2	1.64(m)	25.45
		γCH_2	1.68(m)	29.23
		δCH_2	3.02(t)	41.93
		COO^-		185.28
9	Hypoxanthine	CH	8.20(s)	148.39

		CH	8.22(s)	144.76
10	Alanine	CH	3.79(q)	53.39
		CH_3	1.48(d)	18.97
		COO^-		178.81
11	Choline	CH_3	3.21(s)	56.61
12	Creatine	CH_2	3.93(s)	56.61
		CH_3	3.04(s)	40.21
13	Aspartate	αCH	3.91(m)	55.08
		βCH_2	2.82(m)	39.53
		βCH_2	2.69(m)	39.53
		COO^-		180.28
14	DMA[C]	CH_3	2.76(s)	39.17
15	Formate	$HCOO^-$	8.46(s)	173.9
16	Fumarate	CH	6.52(s)	nd[b]
17	Glutamine	αCH	3.79(m)	57.11
		βCH_2	2.14(m)	29.66
		γCH_2	2.46(m)	33.37
		C=O		180.51
18	Glycine	CH_2	3.57(s)	44.28
		COO^-		175.11
19	Lactate	CH	4.11(m)	71.86
		CH_3	1.33(d)	22.23
		COO^-		185.05
20	Leucine	αCH	3.74(t)	56.39
		βCH_2	1.69(m)	42.68
		γCH_2	1.72(m)	29.26
		CH_3	0.96(d)	23.78
		CH_3	0.97(d)	24.78
		COO^-		177.22
21	Proline	αCH	4.14(dd)	63.96
		βCH_2	2.36(m)	31.82
		βCH_2	2.07(m)	31.82
		γCH_2	2.01(m)	26.73
		δCH_2	3.34(m)	48.92
		δCH_2	3.42(m)	48.92
		COO^-		nd
22	Methionine	αCH	3.87(m)	56.57
		βCH_2	2.19(m)	33.24
		γCH_2	2.65(t)	31.52
		δCH_3	2.14(s)	16.61
		COO^-		nd
23	N-acetyl-glycoproteins	CH_3	2.06(s)	24.83
24	Phenylalanine	αCH	4.00(m)	58.72
		βCH_2	3.29(dd)	39.24

		βCH$_2$	3.13(dd)	39.24
		2CH,6CH	7.43(t)	131.72
		4CH	7.38(m)	130.41
		3CH,5CH	7.33(dd)	132.03
		C(ring)		137.85
		COO$^-$		176.81
25	Propionate	CH$_3$	1.06(t)	12.97
		CH$_2$	2.19(q)	33.22
		COO$^-$		186.72
26	TMA	CH$_3$	2.88(s)	47.24
27	Tyrosine	αCH	3.94(m)	58.96
		βCH$_2$	3.06(m)	38.34
		βCH$_2$'	3.20(m)	38.34
		2CH,6CH	7.20(d)	133.43
		3CH,5CH	6.90(d)	118.56
		1C		157.63
		4C		129.52
		COO$^-$		nd
28	Uracil	CH	5.81(d)	103.51
		CH	7.54(d)	146.22
		C=O		156.59
		C=O		nd
29	Uridine	6CH(ring)	7.88(d)	nd
		5CH(ring)	5.91(d)	92.86
		1CH(ribose)	5.92(d)	nd
		CH$_2$ (ribose)	3.90(m)	nd
		3CH(ribose)	4.23(t)	nd
		2CH(ribose)	4.26(m)	nd
30	Taurine	CH$_2$-SO$_3$H	3.27(t)	50.44
		CH$_2$-NH$_2$	3.43(t)	48.96
31	α-Ketoisovalerate	CH$_3$	1.13(d)	20.09
		CH	3.01(m)	40.3
		αCH	4.00(dd)	58.84
		COO$^-$		176.81
32	α-Glucose	1CH	5.24(d,3.73)	94.76
		2CH	3.54(dd,9.89,3.62)	74.35
		3CH	3.73(dd)	75.42
		4CH	3.42(dd)	72.39
		5CH	3.84(m)	74.38
		CH$_2$	3.85(dd)	63.59
33	β-Glucose	1CH	4.65(d,7.59)	98.76
		2CH	3.25(t)	77.07
		3CH	3.48(t)	78.84
		5CH	3.48(t)	78.84

		CH_2	4.06(m)	63.9
		$CH_{2'}$	3.76(dd)	63.9
34	α-D-GlcNAc	1CH	5.21(d,3.42)	93.77
		2CH	3.88(m)	56.83
		4CH	3.86(m)	74.61
		CH_3	2.06(s)	25.03
35	β-D-GlcNAc	1CH	4.72(d)	97.94
		CH_3	2.06(s)	25.03
36	α-D-Ribose	1CH	5.39(d)	99.36
		2CH	4.12(dd)	nd
		halfCH_2	3.73(dd)	nd
37	β-D-Ribose	1CH	5.26(d)	103.79
		2CH	4.01(m)	nd
		halfCH_2	3.69(dd)	nd
38	α-D-Galactose	1CH	5.27(d)	95.2
		2CH	3.81(m)	nd
		Half 2CH	3.74(dd)	nd
39	β-D-Galactose	1CH	4.59(d)	99.37
		2CH	3.50(m)	nd
40	α-D-Xylose	1CH	5.20(d)	94.71
		2CH	3.52(dd)	nd
41	β-D- Xylose	1CH	4.58(d)	99.4
		2CH	3.24(dd)	nd
42	Methylamine	CH_3	2.60(s)	27.44
43	2-Aminoisobutyrate	CH_3	1.48(s)	24.37
		C		nd
		COO^-		nd
44	β-Fucose	6CH_3	1.21(d)	18.39
		5CH	4.20(m)	70
45	α-Fucose	CH_3	1.25(d)	18.26
		5CH	3.81(m)	74.16
		1CH	5.28(d)	94.88
46	*Allo*threonine	CH_3	1.18(d)	22.05
		CH-OH	4.28(m)	70.75
		CH-NH_2	3.87(dd)	63.15
47	Ethanol	CH_2	1.19(t)	nd
		CH_3	3.66(q)	nd
48	Cytidine	4CH	5.91(d)	nd
		3CH	4.31(dd)	nd
		2CH	6.06(d)	nd
		1CH	7.85(d)	nd
49	Bile acid	CH_3	0.60-0.70(s)	nd
50	Niacin	2CH	8.94(s)	nd
		5CH	7.53	nd

		4CH	8.26	nd
		6CH	8.61	nd
51	UDPG	1CH(gluco ring)	5.62(dd)	nd
52	AMP	2CH	8.61(s)	nd
		7CH	8.26(s)	nd
		1CH'	6.14(d)	nd
		5CH'	4.50(m)	nd
		7CH'	4.05(m)	nd
53	p-Cresol	3 or 5-CH	6.83(d)	117.88
		2 or 6-CH	7.15	nd
54	4-HPA	2, 6CH	7.68(d)	131.94
		3, 5CH	6.87(d)	118.34
55	Histidine	3CH	7.90(m)	nd
		5CH	7.86(m)	nd
		αCH	3.99(m)	nd
		HalfβCH_2	3.13(m)	nd
		HalfβCH_2	3.27(m)	nd
56	4-Hydroxyphenylactate	2, 6CH	7.16(d)	nd
		3, 5CH	6.86(d)	nd
57	Tryptophan	8CH(indole)	7.19(m)	nd
		9CH(indole)	7.29(m)	nd
		6CH(indole)	7.54(d)	nd
		7CH(indole)	7.74(d)	nd
58	Methanol	CH_3	3.36(s)	52.1
59	1-Methylnicotinamide	2CH	9.29(s)	nd
		4CH	8.97(d)	nd
		6CH	8.91(dt)	nd
		5CH	8.19(dd)	nd
		CH_3	4.48(s)	nd

[a] represent the multiplicity: s, singlet; d, doublet; t, triplet; q, quartet; m, multiplet; dd, doublet.
of doublet. [b]nd means not detected. [C]DMA: Dimethylamine; TMA: Trimethylamine; UDPG: Uridine diphosphate glucose; AMP: Adenosine monophosphate; D-GlcNAc: N-Acetyl-D-glucosamine; 4-HPA: 4-hydroxyphenylacetate.

Supplementary Table S2 . The composition of fatty acids in two groups (control and HFD) at day 28 and day 56. The results are shown as mean (μmol/g dried stool) ± SD.*p<0.05, **p<0.0l, ***p<0.001; c: compare control group at day 28 with day 56, # p<0.05.

	control		HFD	
FAs	day 28	day 56	day 28	day 56
C12:0	0.5 ±0.1	1 ±0.1$^{\#}$	0.4 ±0.1	0.7 ±0.1**^{b}
C14:0	5.5 ±1.2	8.6 ±1.2$^{\#}$	11.4 ±1.2**	14.3 ±1.7**
C15:0	3.6 ±1.0	4 ±0.4	3.7 ±1.0**	4.8 ±0.6**
C16:0&C16:1	121.8 ±22.3	175.9 ±30.6$^{\#}$	287.2 ±22.3**	345.4 ±38.7**
C18:0&C18:1	127 ±12.9	192.1 ±45.6$^{\#}$	436.3 ±37.1*	484.8 ±57.2**
C18:2	14.8 ±4.9	23.2 ±1.3	12 ±4.9**	12.1 ±2.4*
C19:0	1.6 ±0.7	2.6 ±0.3	2.2 ±0.7*	2.6 ±0.4*
C20:0	3.8 ±0.5	4.6 ±0.5$^{\#}$	4.6 ±0.5*	5 ±0.7*
C20:1	2.1 ±0.7	2 ±0.4	2.7 ±0.7*	2.4 ±0.4*
C22:1	3.7 ±0.8	4.2 ±0.2	1.8 ±0.8***	2.1 ±0.3**
C24:0	3.7 ±0.7	4.6 ±0.2$^{\#}$	1.5 ±0.7**	1.8 ±0.3**

Supplementary Table S3. The scores of multivariate statistical analysis compared HFD group with control group at day 0,day 7,day 14,day 28,day 42,day 56, day 60, day 66, day 71, day 76 and day 81 after dietary intervention.

day(s)	PCA		permutation test	OPLS-DA		p value[a]
	R^2X	Q^2		R^2X	Q^2	
0	0.908	0.675	×[b]	0.466	-0.309	1.00E+00
7	0.901	0.828	√	0.746	0.940	5.39E-09
14	0.927	0.866	√	0.793	0.936	6.13E-10
28	0.794	0.649	√	0.676	0.864	5.25E-08
42	0.822	0.627	√	0.742	0.914	2.47E-09
56	0.853	0.747	√	0.766	0.948	2.61E-09
60	0.870	0.746	√	0.842	0.868	7.46E-07
66	0.791	0.486	√	0.749	0.930	3.55E-10
71	0.926	0.836	√	0.769	0.888	2.53E-08
76	0.880	0.775	√	0.732	0.909	1.18E-08
81	0.798	0.646	√	0.712	0.888	8.44E-09

a: p values were generated by CV-ANOVA. b ×: pattern of PLS-DA is not successfully checked by permutation test; √ : pattern of PLS-DA is successfully checked by permutation test.

Supplementary Table S4. Pearson correlation coefficient values for the HFD-induced significant changes of microbial species at day 7, day 28, day 56 and day 81 ($p<0.05$). Red indicates increases and blue decreases.

Species-Bacteria	day7	day28	day56	day81
Acidovorax_wohlfahrtii	-	-	0.854	0.613
Allobaculum_stercoricanis	-	-	0.673	-
Allobaculum_unclassified	-	0.577	-0.721	-
Allobaculum_uncultured_bacterium	-	-0.622	-	-
Allobaculum_uncultured_erysipelotrichales_bacterium	-	-0.751	-	-
Alloprevotella_unclassified	-	-	-0.723	-
Anaerotruncus_sp._G3(2012)	-	-	0.824	-
Anaerotruncus_unclassified	-	-	0.799	-
Anaerotruncus_uncultured_clostridiales_bacterium	-	-	0.734	-
Bacteroidales_unclassified	-	-	-0.792	-
Bacteroides_acidifaciens	-	0.682	-	-
Bacteroides_uniformis	-	-	0.593	-
Bacteroidetes_unclassified	-	-	0.704	-
Blautia_glucerasea	-	-	0.901	-
Blautia_uncultured_bacterium	-	0.763	-	-
Blautia_uncultured_clostridiales_bacterium	-	-	0.665	-
Blautia_uncultured_ruminococcus_sp.	-	-	0.600	-
Burkholderia_uncultured_beta_proteobacterium	-	-	-0.620	-
Butyricimonas_synergistica	-	-	0.625	-
Butyricimonas_uncultured_bacterium	-	-	0.653	-
Butyricimonas_virosa	-	-	0.756	-
Carnobacterium_maltaromaticum	-	-0.812	-	-
Christensenellaceae_uncultured_bacteroidetes_bacterium	-	-0.595	-0.693	-0.647
Clostridium_papyrosolvens	-	-	0.816	-
Clostridium_sensu_stricto_1_unclassified	-	-0.807	-0.842	-0.654
Clostridium_sp._Clone-17	-	-	-	0.602
Clostridium_sp._Culture-27	-	-0.616	-	-
Comamonadaceae_unclassified	-	-	0.945	-
Coprococcus_uncultured_bacterium	-	-	0.834	-
Coprococcus_uncultured_firmicutes_bacterium	-	-	0.829	-
Desulfovibrio_unclassified	-	-	0.676	-
Dysgonomonas_unclassified	-	-	-0.622	-
Enterococcus_gallinarum	-	-	0.625	-
Erysipelotrichaceae_uncultured_organism	-	-	0.704	-0.553
Eubacterium_sp._14-2	-	-	0.601	-
Family_XIII_unclassified	-	-	-0.717	-
Family_XIII_uncultured_bacterium	-	-	-0.671	-
Family_XIII_uncultured_clostridiales_bacterium	-	-	0.774	-
Flavobacteriaceae_unclassified	-	0.608	-	-
Intestinimonas_uncultured_bacterium	-	-	0.964	-

Jeotgalicoccus_uncultured_bacterium	-	-	0.709	-
Lachnospiraceae_bacterium_3_1_57FAA_CT1	-	-	0.809	-
Lachnospiraceae_uncultured_bacterium	-	0.920	0.940	0.810
Lachnospiraceae_uncultured_clostridiales_bacterium	-	-	0.764	0.565
Lactococcus_plantarum	-	-	0.798	-
Marvinbryantia_uncultured_bacterium	-	-	0.611	-
Microbacterium_maritypicum	-	0.583	0.887	-
Mycobacterium_unclassified	-	-	-	0.554
Oscillibacter_uncultured_clostridiales_bacterium	-	-	0.859	0.592
Parabacteroides_distasonis	-	-	-	-0.632
Parabacteroides_distasonis_ATCC_8503	-	0.593	-	-
Parabacteroides_unclassified	-	0.582	0.993	-
Parasutterella_secunda	-	-	-0.830	-
Parvibacter_unclassified	-	-	0.915	-
Parvibacter_uncultured_bacterium	-	-0.593	-	-
Peptococcus_uncultured_bacterium	-	-	-	0.581
Prevotellaceae_uncultured_bacterium	-	-0.699	-	-
Pseudomonas_unclassified	-	-	0.776	-
RF9_uncultured_bacterium	-	-0.754	-0.734	-
Roseburia_uncultured_bacterium	-	0.804	0.922	0.814
Rothia_unclassified	-	-	0.823	0.726
Ruminococcaceae_uncultured_bacterium	-	-0.655	-0.643	-0.573
Ruminococcaceae_uncultured_bacterium_adhufec311	-	-	0.688	-
S24-7_uncultured_bacterium	-	-0.604	-0.745	-0.613
S24-7_uncultured_bacteroidales_bacterium	-	-	-0.882	-
Slackia_unclassified	-	-	0.641	-
Thalassospira_uncultured_organism	-	0.600	-	-
Turicibacter_sp._LA61	-	-0.696	-	-
ratAN060301C_uncultured_bacterium	-	-	0.672	-
unidentified_rumen_bacterium_12-110	-	-	0.901	0.650
unidentified_rumen_bacterium_12-124	-	-0.663	-0.809	-0.676
unidentified_rumen_bacterium_JW32	-	-0.723	-	-

Supplementary Table S5. Correlations between the HFD-induced changes of fecal bile acids and of microbes in the family, genus and species levels (p-values were Pearson correlation coefficients with red ones indicating positive correlations whereas the blue ones indicating negative correlations).

	bile acids
family	
Deferribacteraceae	-0.255
Defluviitaleaceae	-0.263
Porphyromonadaceae	-0.384
Ruminococcaceae	0.257
Bacteroidales_unclassified	-0.297
Unknown_Family	0.261
genus	
Anaerotruncus	-0.258
Blautia	-0.43
Burkholderia	0.282
Caulobacter	0.323
Coprobacillus	-0.245
Coprococcus	-0.332
Escherichia-Shigella	-0.313
Intestinimonas	-0.421
Oscillibacter	-0.313
Parabacteroides	-0.299
Roseburia	-0.409
Pseudobutyrivibrio	-0.325
Bacteria_unclassified	0.344
Christensenellaceae_uncultured	0.341
Defluviitaleaceae_unclassified	-0.345
Erysipelotrichaceae_uncultured	-0.262
Family_XIII_unclassified	0.375
Lachnospiraceae_norank	-0.34
Lachnospiraceae_unclassified	-0.397
Lachnospiraceae_uncultured	-0.431
Peptostreptococcaceae_incertae_sedis	0.244
Prevotellaceae_unclassified	0.376
RF9_norank	0.468
Ruminococcaceae_unclassified	0.318
Ruminococcaceae_uncultured	0.397
species	
Acidovorax_wohlfahrtii	-0.327
Allobaculum_stercoricanis	-0.387
Allobaculum_uncultured_erysipelotrichales_bacterium	0.255

Anaerotruncus_sp._G3(2012)	-0.355
Anaerotruncus_unclassified	-0.442
Anaerotruncus_uncultured_clostridiales_bacterium	-0.319
Bacteroidales_unclassified	0.343
Bacteroides_uniformis	-0.339
Bacteroidetes_unclassified	-0.255
Blautia_glucerasea	-0.358
Blautia_uncultured_bacterium	-0.273
Blautia_uncultured_clostridiales_bacterium	-0.309
Blautia_uncultured_ruminococcus_sp.	-0.250
Burkholderia_uncultured_beta_proteobacterium	0.384
Butyricimonas_uncultured_bacterium	-0.246
Butyricimonas_virosa	-0.295
Christensenellaceae_uncultured_bacteroidetes_bacterium	0.308
Clostridium_papyrosolvens	-0.307
Clostridium_sensu_stricto_1_unclassified	0.392
Clostridium_sp._Culture-27	0.363
Comamonadaceae_unclassified	-0.379
Coprococcus_uncultured_bacterium	-0.345
Coprococcus_uncultured_firmicutes_bacterium	-0.323
Enterococcus_gallinarum	-0.317
Erysipelotrichaceae_uncultured_organism	-0.308
Family_XIII_uncultured_bacterium	0.329
Family_XIII_uncultured_clostridiales_bacterium	-0.348
Intestinimonas_uncultured_bacterium	-0.382
Jeotgalicoccus_uncultured_bacterium	-0.393
Lachnospiraceae_bacterium_3_1_57FAA_CT1	-0.385
Lachnospiraceae_uncultured_bacterium	-0.423
Lachnospiraceae_uncultured_clostridiales_bacterium	-0.354
Lactococcus_plantarum	-0.371
Marvinbryantia_uncultured_bacterium	-0.258
Microbacterium_maritypicum	-0.387
Oscillibacter_uncultured_clostridiales_bacterium	-0.297
Parabacteroides_unclassified	-0.452
Parasutterella_secunda	0.270
Parvibacter_unclassified	-0.411
Parvibacter_uncultured_bacterium	0.330
Prevotellaceae_uncultured_bacterium	0.375
Pseudomonas_unclassified	-0.325
RF9_uncultured_bacterium	0.443
Roseburia_uncultured_bacterium	-0.397
Rothia_unclassified	-0.312
Ruminococcaceae_uncultured_bacterium	0.415
Ruminococcaceae_uncultured_bacterium_adhufec311	-0.268

Thalassospira_uncultured_organism	-0.259
Turicibacter_sp._LA61	0.331
ratAN060301C_uncultured_bacterium	-0.440
unidentified_rumen_bacterium_12-110	-0.361
unidentified_rumen_bacterium_12-124	0.362
unidentified_rumen_bacterium_JW32	0.251

Supporting information for materials and methods

Chemicals

Deuterium oxide (D_2O, 99.9 atom %D), Methyl tricosanate (99.0%), methyl heptadecanoate (99.0%) and acetyl chloride were purchased form Sigma-Aldrich Inc. (St. Louis, MO) and sodium 3-trimethylsilyl [2,2,3,3-$^{2}H_4$] propionate (TSP) was bought from Cambridge Isotope Laboratories, Inc. (Tewksbury, MA,USA). Sodium azide (NaN_3) was bought from Fuchen Chemical Reagent Company (Tianjin, China) whilst K_2CO_3, $K_2HPO4 \cdot 3H_2O$, $NaH_2PO_4 \cdot 2H_2O$ were from Sinopharm Chemical Co. Ltd (Shanghai, China). A mixed standard of 37 fatty acid methyl esters and 3,5-ditertbutyl-4-hydroxytoluene (BHT) were obtained from Supelco (Bellefonte, PA).

Sample Preparation for NMR and GC-FID/MS Analysis

Each fecal sample (about 60 mg) was individually extracted by vortex mixing with 600 μL phosphate buffer followed with freeze-thaw treatment thrice and homogenization with tissuelyser (QIAGEN, Hilden, Germany) at 20 Hz for 90s. The supernatants were obtained after 10 mins centrifugation (16000 x *g*, 4℃). After repeating this extraction of the residues once, two supernatants from each sample were pooled together and centrifuged for another 10 mins (16000 x

g, 4℃). 600 μL supernatant from each sample was individually transferred into a 5 mm NMR tube for analysis.

For fatty acid methylation, about 2 mg feces sample was homogenized with 0.1 mL methanol in a Pyrex tube followed with freeze-thaw thrice. 1 mL mixed solvent of methanol and hexane (4:1; v/v) was added into the above mixture together with 20 μL internal standards (containing 0.2mg/ml of BHT, 0.05mg/mL methyl tricosanate and 0.1mg/mL methyl heptadecanoate). The mixture were cooled and subjected to acetyl chloride catalyzed methylation for 24 hours in the dark (ambient temperature was 25℃). Methyl esters of fatty acids extracted with hexane thrice followed with extract combination, removal of solvent and reconstitution into 50 μL hexane before GC-FID/MS analysis.

Analysis of Fatty Acids with GC-FID/MS

The measurement of fatty acids of fecal samples was done on a Shimadzu GCMS-QP2010Plus spectrometer (Shimadzu Scientific Instruments) as reported previously[27,35] with some minor modifications. The temperature of injection port and flame ionization detector (FID) were both set to 230℃. The temperature gradient started with 45℃ for 1 min and then was increased to 195℃ (30℃/min). After holding at 195℃ for 3 mins, temperature was raised to 230℃ /min with a rate of 7℃/min and kept for 1 min. The methylated fatty acids in fecal samples were identified by comparing their retention times and mass spectrometry data with a mixed standard of methylated fatty acids whilst quantified using internal standards. The levels of fatty acids were expressed as μmol fatty acids per gram of dry fecal sample.

NMR Analysis of Fecal Extracts

All NMR spectra were acquired at 298 K on a Bruker AVANCE III 600 MHz NMR

spectrometer having an inverse cryogenic probe (Bruker Biospin, Germany) with ^{1}H and ^{13}C frequencies of 600.13 and 150.90 MHz, respectively. One-dimensional ^{1}H NMR spectra were acquired using standard noesygppr1d pulse sequence with the recycle delay of 2 s, mixing time of 100 ms and acquisition time of 1.36 s. 90° pulse width was adjusted to about 10 μs for each sample and 128 transients were collected into 32 000 data points over a spectral width of 20 ppm. For resonance assignments, a set of two-dimensional NMR spectra was acquired for some selected samples including ^{1}H J-resolved spectroscopy (^{1}H JRES), ^{1}H–^{1}H total correlation spectroscopy (TOCSY), ^{1}H–^{1}H correlation spectroscopy (COSY), ^{1}H–^{13}C heteronuclear single quantum correlation (HSQC) and ^{1}H–^{13}C heteronuclear multiple bond correlation (HMBC) spectra.

NMR Data Processing and Multivariate Data Analysis

All FIDs obtained from one-dimensional NMR experiments were multiplied with an exponential window function with a line-broadening factor of 1Hz prior to Fourier transformation. All spectra were corrected for phase and baseline using the software TOPSPIN (V3.0, Bruker Biospin, Germany) with chemical shift referenced to the methyl signal of TSP (δ 0.00). The spectral region of δ 0.5-9.5 was then bucketed into bins with the width of 0.004 ppm (2.4 Hz) using AMIX software (V3.8.3, Bruker Biospin) and the region of δ 4.68-5.15 was discarded to eliminate the distorted water signal. The integrated areas of all bins were normalized to the dry sample weights so that the resultant data represented the absolute concentration of bins (or metabolites) in the form of peak area per milligram dry sample.
